# Primary cilia and neural computation

**DOI:** 10.1186/s12929-026-01264-9

**Published:** 2026-06-02

**Authors:** Andrew Teoh, Mishra Arinjay, Dwaipayan Giri, Siew Cheng Phua

**Affiliations:** 1 Department of Biological Sciences, National University of Singapore, Singapore, 117558 Singapore; 2 Department of Biology, Indian Institute of Science Education and Research (IISER), Pune, Maharashtra 411008 India

**Keywords:** Ciliopathies, G-protein coupled receptors (GPCRs), Memory, Neural computation, Neuromodulation, Neurodevelopmental disorders, Plasticity, Primary cilia, Signal compartmentalization, Synapses

## Abstract

Ciliopathies frequently involve abnormal brain development and affected individuals often present with varying degrees of cognitive impairment and behavioral alterations. In parallel, genetic studies have linked primary cilia to neurodevelopmental disorders such as autism spectrum disorder (ASD). Together, these observations point to a role for primary cilia in cognition, yet their mechanistic contribution to neural function remains unclear. To address this, we define neural computation as the integration of inputs, dynamic thresholding, and routing of outputs, and propose that primary cilia function not as passive sensory antennae, but as dynamic computational microdomains. Within this framework, cilia integrate extrinsic signals and intrinsic cellular states through modular signaling pathways, including GPCR-cAMP-PKA cascades and tightly regulated trafficking mechanisms. These processes are spatially constrained by ciliary gating and transport systems, enabling selective filtering, amplification, and transformation of inputs into context-dependent outputs. During development, these molecular computations scale to shape neural circuit architecture. Ciliary signaling regulates neurogenesis, specifies neuronal identity, and guides neuronal migration and connectivity, thereby embedding computational parameters into the physical structure of the brain. In the mature brain, ciliary GPCRs modulate neuronal and circuit-level dynamics. Receptors such as 5HT6 and SSTR3 influence neuronal excitability and excitation-inhibition balance, while hypothalamic MC4R functions as a rheostat to stabilize state-dependent signaling. In parallel, dynamic trafficking of DRD1 receptors enables flexible regulation of dopaminergic signaling across subcellular compartments. Disruption of ciliary function has been linked to memory impairments, suggesting a role in regulating the stability and competition of memory engrams. These effects may involve multiple plasticity mechanisms, including synaptic tagging and capture, activity-dependent synchronization, and adult neurogenesis. Together, these findings support a unifying view in which primary cilia perform molecular computations that scale across development and adult brain function to influence neural circuits and behavior. Future integration of cilia-targeted molecular tools with systems-level approaches will be essential for disentangling developmental effects from active computational roles in the mature brain.

## Primary cilia and neural computation

### Cognition and primary cilia

Ever since the neuron doctrine became established in neuroscience [1], the computational capacity of the brain has been primarily attributed to synaptic transmission [2] and network connectivity [3]. Individual neurons integrate diverse chemical and electrical inputs within their dendrites to generate outputs in the form of action potentials and neurotransmitter release [4], while interactions across neural circuits further transform these signals into coordinated behavioral responses [5]. Consequently, studies of cognitive function and dysfunction have largely focused on synapses and neuronal circuits [6–10].

Among the many conditions associated with cognitive impairment, ciliopathies represent a prominent class of disorders arising from defects in the primary cilium. These include Bardet-Biedl syndrome (BBS) [11], Joubert syndrome (JS) [12], orofaciodigital syndrome [13], and acrocallosal syndrome [14]. Although clinically heterogeneous, these disorders frequently present with altered brain architecture [15–17], alongside a range of developmental and behavioral abnormalities such as intellectual disability, hyperactivity, anxiety, and behavioral rigidity [16, 18–25]. Beyond classical ciliopathies, genetic studies have identified enrichment of cilia-associated genes in disorders including autism spectrum disorder (ASD), schizophrenia (SCZ), major depressive disorder, and Alzheimer’s disease [26–29]. These observations suggest that primary cilia may play broader roles in shaping brain function than previously appreciated.

The canonical role of the primary cilium is as a signaling compartment for pathways such as Hedgehog (HH) signaling [30], which regulates key developmental processes including progenitor proliferation [31], neuronal differentiation [32], migration [32, 33], and neurite formation [34, 35]. Through these roles, ciliary signaling contributes to the structural organization of the developing brain. Importantly, primary cilia persist in most neurons in the adult brain, where they are enriched with neuromodulatory G-protein coupled receptors (GPCRs) that influence neuronal activity [36–39]. Despite these developmental and post-developmental roles, how primary cilia contribute to neural computation remains poorly understood.

### What is neural computation?

Defining “computation” in a biological context depends on perspective. In molecular biology, computation is often framed in terms of biochemical signaling networks that integrate inputs to regulate cellular decisions [40, 41]. In systems neuroscience, computation is typically defined by how neuronal activity encodes and transforms information across specific neural circuits [42, 43]. To bridge these views, we define neural computation as a process involving three core operations: (i) integration of inputs, (ii) discretization through thresholding, and (iii) routing of outputs. Neural systems continuously receive analogue inputs, such as graded membrane potentials and neuromodulatory signals, which must be integrated and transformed into discrete outputs that drive physiological or behavioral responses [44, 45]. Importantly, these outputs are context-dependent: identical inputs can produce different outcomes depending on the internal state of the system, including prior activity, molecular composition, and network configuration.

Across scales, neural computation also involves operations such as signal filtering, amplification, and the storage and retrieval of information [46–48]. These processes enable the nervous system to extract relevant information from noisy inputs and generate adaptive responses.

### Physical correlates of neural computation

Neural computation is implemented across multiple physical scales. At the molecular level, ligand-receptor interactions regulate signaling cascades, such as GPCR-mediated activation of adenylate cyclases [36]. Certain molecular systems function as conditional logic gates; for example, NMDA receptors require coincident binding of glutamate and glycine for activation [49].

At the subcellular level, distinct neuronal compartments perform specialized computational operations. Synapses integrate inputs and mediate activity-dependent plasticity [50], dendrites shape signal propagation through nonlinear transformations [51–53], and the axon initial segment (AIS) determines whether integrated inputs reach the threshold for action potential generation [54–59]. These compartments collectively implement operations analogous to filtering, gain control, and thresholding.

At larger scales, neural circuits integrate competing inputs over time to produce coordinated outputs. For example, hypothalamic circuits regulate feeding behavior by integrating fast synaptic inputs with slower neuromodulatory signals [60–66], generating graded behavioral states. Across the brain, distributed networks further support higher-order processes such as reinforcement learning and decision making through coordinated activity between regions such as the cortex, striatum, and hippocampus [67–70]. A key principle across these scales is the dynamic regulation of thresholds, often governed by excitation-inhibition (E/I) balance, which is essential for stable and flexible computation [71–76].

With a synapse-heavy narrative dominating neurobiology, the potential contribution of other subcellular structures, including the primary cilia, to neural computation remains relatively less well understood.

### Positioning primary cilia within neural computation

Primary cilia possess several features that position them as potential contributors to neural computation. As specialized signaling compartments, they concentrate diverse receptors, including GPCRs, within a confined space, enabling the detection and integration of multiple extracellular cues [77]. Their signaling output is further shaped by tightly regulated trafficking [78, 79] and gating [80] mechanisms that control the composition of ciliary proteins, as well as by structural dynamics [81] that can modulate signaling capacity.

Through these properties, primary cilia are well positioned to influence how signals are filtered, integrated, and transformed before contributing to neuronal output. By integrating extrinsic inputs with intrinsic cellular states, cilia may generate context-dependent signaling outputs that affect neuronal function and circuit dynamics. These features raise the possibility that primary cilia act not merely as passive signaling antennae, but as subcellular compartments that actively participate in neural computation.

In this review, we examine the potential role of primary cilia as computational microdomains across multiple scales of neural organization. Sect. "Primary cilia as molecular computational microdomains" discusses how the structural and molecular properties of the primary cilium enable signaling processes that resemble fundamental computational operations. Sect. "Primary cilia shape neural computation through brain development" examines how these processes influence brain development by shaping neuronal number, identity, positioning, and connectivity. Sect. "How ciliary GPCRs influence computational processes in the adult brain" explores how ciliary GPCR signaling modulates neuronal properties and circuit dynamics in the adult brain. Finally, Sect. "An emerging role of primary cilia and brain memory" considers emerging evidence linking ciliary signaling to memory and higher-order cognitive processes. Together, these perspectives provide a unified framework for understanding how primary cilia contribute to neural computation from molecules to behavior.

## Primary cilia as molecular computational microdomains

Most cell types possess a primary cilium at some stage of their development. While the core molecular machinery underlying cilium assembly and maintenance, such as intraflagellar transport (IFT), the BBSome, and the Transition Zone (TZ), is largely conserved, the precise molecular composition of primary cilia varies substantially across cell types and cellular states. A diverse repertoire of signaling receptors is selectively enriched within cilia. These include GPCRs such as SMO, GPR161, 5HT6, DRD1, MCHR1, and SSTR3, which have been extensively reviewed elsewhere [82, 83]. In addition, receptor tyrosine kinases [84], including IGF1R [85] and PDGFRα [86, 87], localize to primary cilia and initiate downstream signaling cascades through ligand-induced phosphorylation. Cilia also harbor specific TRP ion channels [88], which mediate calcium influx in response to mechanical and chemical stimuli. These receptors and channels converge on a set of ciliary-localized enzymatic effectors, including adenylate cyclase III (AC3) [89], INPP5E [90, 91], protein kinase A (PKA) [92], and transcriptional regulators such as GLI2/3 [93]. Importantly, these signaling pathways can operate independently or engage in extensive crosstalk, giving rise to integrated and context-dependent signaling outputs.

Biological systems continuously process information to generate appropriate cellular responses. At the molecular level, this process can be conceptualized as molecular computation, in which networks of interacting biomolecules transform inputs into outputs through defined biochemical operations [40, 41]. These operations include the detection of external and internal signals, their integration across multiple pathways, and their transformation into context-dependent outputs that influence cellular behavior. Importantly, such transformations are often nonlinear and state-dependent, meaning that identical inputs can produce distinct outputs depending on the molecular composition, spatial organization, and prior activity of the system [94]. Within cells, these computational processes are frequently constrained to specialized subcellular compartments that organize signaling components in space and time. By controlling the localization, concentration, and interaction dynamics of receptors, enzymes, and second messengers, these compartments enable selective filtering of inputs, amplification or attenuation of signals, and integration across modalities [95].

**Fig. 1:**
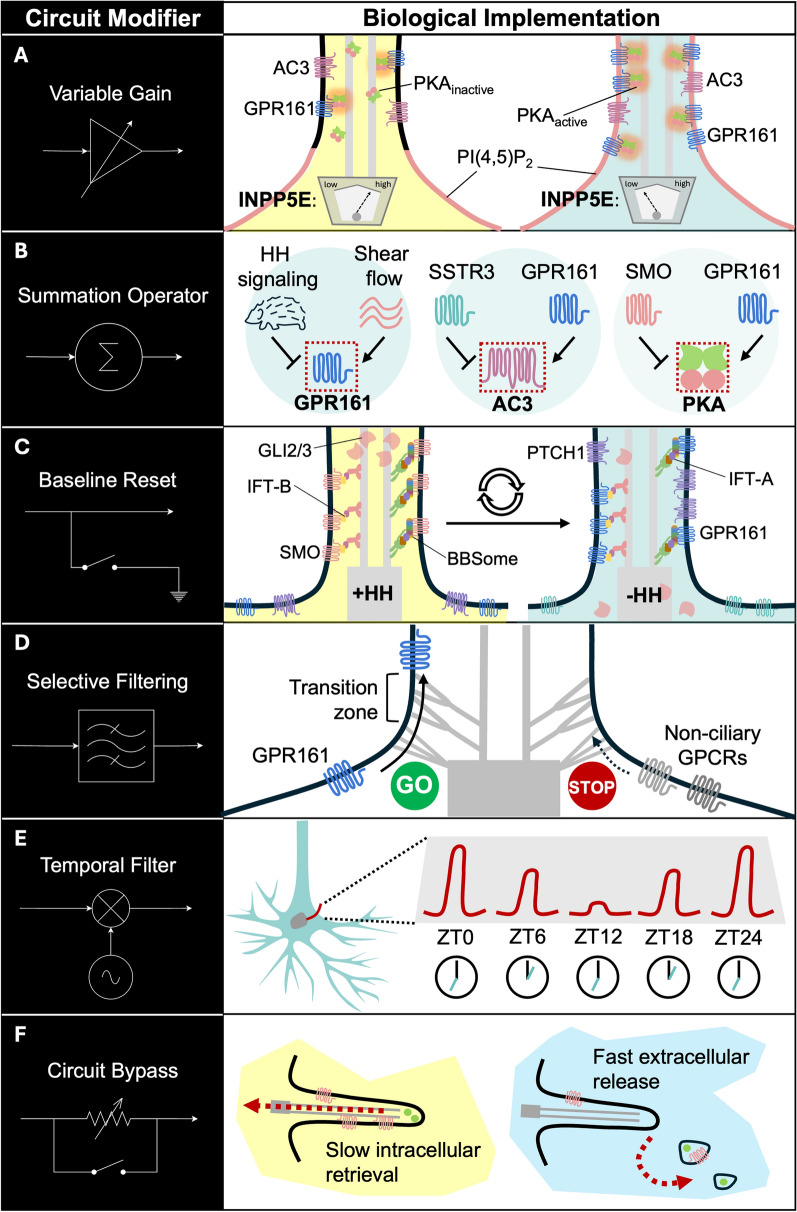
The computational toolbox of primary cilia signaling. Primary cilia use distinct structural and molecular features to execute discrete information-processing operations, analogous to electronic circuit modifiers (left column), through specific biological mechanisms (right column). **A** Variable gain. As illustrated by the variable amplifier symbol (left), ciliary signaling amplitude can be dynamically tuned by regulating the abundance of signaling components within the cilium. INPP5E activity modulates PI(4,5)P_2_-dependent trafficking of GPR161 into the ciliary membrane, thereby controlling ciliary GPR161 levels. In turn, ciliary GPR161 contributes to local PKA activity through AC3-generated cAMP. **B** Summation operator. Signal integration, illustrated by a general summation operator symbol (left), is achieved through the convergence of multimodal or parallel inputs onto shared effector nodes (right). This occurs at multiple levels of the HH signaling cascade: physical cues such as shear flow and biochemical HH signals converge on GPR161; Gs-coupled GPR161 and Gi-coupled SSTR3 converge on AC3 to regulate cAMP production; and activated SMO and GPR161 exert opposing influences on PKA to determine net PKA-dependent output. **C** Baseline reset. Baseline resetting, illustrated by a switch to ground state (left), is mediated by coordinated receptor influx and efflux through IFT and the BBSome (right). Upon HH pathway activation, IFT and the BBSome mediate the removal of SMO and GLI from cilia while promoting the return of PTCH1 and GPR161 to the cilium. This terminates ongoing HH signal transduction and restores sensitivity to future HH signals. **D** Selective filtering. Selective filtering, illustrated by a bandpass filter (left), is mediated by the transition zone, which acts as a selective diffusion barrier (right). This physical gate isolates the ciliary compartment by permitting entry of ciliary receptors, such as GPR161, while excluding non-ciliary GPCRs, thereby reducing cytosolic noise and maintaining a privileged signaling space. **E** Temporal filter. Temporal filtering, illustrated by a signal multiplier symbol together with temporal fluctuations represented by a sinusoidal current source (left), is mediated by circadian-driven cycles of ciliary assembly and disassembly (right). These cycles allow ciliary length to vary as a function of time of day, represented by zeitgeber time (ZT). Such rhythmic structural plasticity may act as an overarching temporal filter, selectively enhancing or attenuating specific neuronal signals according to circadian physiological demands. **F** Circuit bypass. Circuit bypass, illustrated by a parallel bypass shunt (left), is mediated by actin-dependent ciliary decapitation (right). This process enables the acute extracellular removal of signaling receptors and effectors from primary cilia and may represent a rapid alternative to slower intracellular retrieval mediated by IFT and the BBSome

In this framework, primary cilia can be viewed not merely as sites of signal reception, but as structured biochemical microdomains capable of performing molecular computations through coordinated signaling, trafficking, and structural mechanisms (Fig. 1). In the following sections, we leverage the well-characterized HH signaling pathway to illustrate how signaling components are modularly organized within primary cilia and how structural plasticity may further expand the versatility of these signaling architectures. Through these examples, we argue that primary cilia do not merely passively decode extracellular cues, but function as specialized subcellular compartments that perform complex molecular computations, integrating both extrinsic signals and intrinsic cellular states to shape downstream responses.

### The modular organization of primary cilia signaling enables versatile molecular computation

Much of our current understanding of primary cilia signaling derives from studies of the HH pathway. In its canonical depiction, binding of HH ligand to PTCH1 within the primary cilium triggers the exit of PTCH1 and the entry of SMO. Activated SMO then promotes the accumulation of transcription factors GLI2/3 at the ciliary tip, facilitating their processing into activator forms that subsequently translocate to the nucleus to regulate gene expression [30, 96–98]. While this framework captures the core logic of HH signaling, it represents only a simplified view of a far more complex and modular signaling architecture in which multiple regulatory layers govern signal propagation, amplification, and integration, as detailed below.

Genetic and proteomic studies have revealed an expanding network of regulatory components that refine HH signaling within the cilium. At basal state, the GPCR GPR161 is trafficked into primary cilia via a TULP3-dependent mechanism [99, 100]. GPR161 activates AC3, leading to cAMP production and PKA activation [99]. PKA phosphorylates GLI3, promoting its processing into the GLI3R repressor form, thereby suppressing HH target gene expression [101, 102]. In this manner, GPR161 functions as a key antagonist of HH signaling under basal conditions [99, 100], effectively imposing a tonic inhibitory bias and ensuring that pathway activation requires sufficiently strong upstream input.

GPR161 also acts as an A-kinase anchoring protein (AKAP) for type I PKA regulatory subunits (RI), enabling the formation of a spatially confined cAMP-PKA signaling module within the cilium [103]. This arrangement allows locally generated cAMP to selectively activate cilia-associated PKA, illustrating a form of signal compartmentalization, in which biochemical signals are restricted to defined microdomains to preserve specificity and prevent cross-activation. Moreover, GPR161 integrates lipid signaling into the HH pathway [100]. The phosphoinositide composition of the ciliary membrane is tightly regulated by INPP5E, which converts PI(4,5)P_2_ to PI(4)P (90,91,100). TULP3 binds PI(4,5)P_2_ and mediates the trafficking of GPR161 into cilia. Consequently, loss of INPP5E elevates ciliary PI(4,5)P_2_ levels, leading to increased ciliary recruitment of TULP3 and GPR161 and enhanced repression of HH signaling [100]. Thus, ciliary phosphoinositide composition functions as a modulatory layer that tunes pathway output via control of GPCR localization. This acts as a variable gain mechanism, where membrane lipid composition dynamically scales the input–output response of the signaling system (Fig. 1A).

Emerging evidence further expands the functional repertoire of GPR161. Recent studies suggest that GPR161 is mechanosensitive and can respond to fluid shear stress to regulate neuronal saltatory migration in the early postnatal brain via a PKA-NDE1 pathway [104]. This finding illustrates how canonical signaling components can be repurposed to integrate distinct physical cues within the ciliary compartment, analogous to multimodal input integration, where biochemical and mechanical signals converge onto shared downstream effectors, in this case being GPR161 (Fig. 1B).

Beyond GPR161, additional regulators further highlight the modularity of ciliary HH signaling. GPCR kinase 2 (GRK2) transiently accumulates in primary cilia to phosphorylate and activate SMO, which in turn can bind and sequester PKA catalytic subunits to restrict GLI processing [105]. In this way, GPR161 and SMO exert opposing control over ciliary PKA through distinct mechanisms: GPR161 enhances local PKA activation by bringing PKA into proximity with sites of cAMP production, whereas SMO constrains PKA-dependent GLI processing by altering catalytic subunit availability. Ciliary PKA therefore functions as a specialized integrative node, converting the balance between local second-messenger production and substrate accessibility into a graded HH signaling output (Fig. 1B).

Crosstalk with calcium signaling provides yet another layer of integration. In mouse embryonic fibroblasts and IMCD3 cells, HH pathway activation elevates ciliary Ca^2^⁺ levels [106]. In *Xenopus laevis* embryos, HH signaling specifically activates TRPC3 channels, promoting Ca^2^⁺ release from intracellular stores [107]. This Ca^2^⁺ signaling axis antagonizes proliferative Sonic HH responses in Neural Stem Cells (NSCs) by downregulating SOX2 and upregulating neurogenic genes, thereby promoting neuronal differentiation [107]. Here, Ca^2^⁺ signaling acts as a context-dependent switch, redirecting HH pathway output toward alternative cellular fates depending on intracellular state.

Finally, ciliary GPCRs that regulate cAMP levels can interface directly with the HH pathway. For example, activation of the Gi-coupled receptor SSTR3 reduces AC3 activity and cAMP levels [108]. In mouse fibroblasts, SSTR3 activation is sufficient to enhance GLI1 expression [108], likely through reduced PKA activity. This underscores how multiple GPCR inputs can converge on shared second messengers to modulate HH signaling output. Here, the shared enzymatic effector AC3 serves as a canonical example of parallel signal integration, combining inputs from distinct GPCRs to generate a net, localized cAMP output (Fig. 1B).

Collectively, these findings reveal that primary cilia organize signaling components into modular, interacting units, namely GPCR-cAMP-PKA modules, lipid-dependent trafficking modules, and Ca^2^⁺ signaling modules, that can be independently regulated yet dynamically coupled. Such an architecture enables primary cilia to perform operations analogous to thresholding, gain control, feedback regulation, and multimodal integration, supporting the view that they function as specialized compartments for molecular computation rather than simple signal decoders.

### Ciliary trafficking and gating enable molecular computation through spatiotemporal organization

The molecular computations described above are enabled by the precise spatial and temporal positioning of signaling components within the primary cilium. The orchestrated entry, exit, and retention of these molecules are governed by specialized trafficking and gating mechanisms that collectively define the ciliary signaling landscape. By controlling when and where specific receptors and effectors are present, these systems establish the physical framework within which signaling interactions and hence molecular computations can occur (Fig. 1).

IFT constitutes the core trafficking machinery of the primary cilium. This bidirectional motor system operates along the axonemal microtubules, with the IFT-B complex associating with kinesin-2 motors to drive anterograde transport toward the ciliary tip, and the IFT-A complex coupling to dynein-2 to mediate retrograde transport back to the cell body [78]. In addition to ciliogenesis, IFT plays a continuous role in regulating protein turnover within the cilium. Genetic studies have revealed functional specialization within the IFT system: certain components, such as IFT88 [109], are indispensable for cilium assembly, whereas others selectively regulate the trafficking of signaling molecules without disrupting ciliogenesis. For example, IFT25 and IFT27, both components of the IFT-B complex, are not required for ciliogenesis but are critical for HH signaling [110, 111]. While IFT25 regulates the removal of PTCH1 upon ligand binding [110], both IFT25 and IFT27 restrict SMO entry into cilia at basal state, and facilitate the accumulation of GLI2 at the ciliary tip during pathway activation [110, 111]. Similarly, IFT38 interacts with the BBSome (see paragraph below) to mediate the export of GPR161 across the TZ [112]. In contrast, disruption of IFT-A components such as IFT139 [113] or IFT43 [114] leads to aberrant accumulation of SMO and GLI proteins in cilia under basal conditions, reflecting defects in retrograde trafficking and protein redistribution. Collectively, these findings indicate that the integrity of IFT complexes is required to establish the correct balance of receptors and effectors within the cilium, thereby setting the baseline conditions for signal responsiveness, and resetting the baseline upon signal transduction (Fig. 1C). In this context, IFT functions analogously to a targeted routing system, ensuring that signaling components are delivered to, or removed from, specific ciliary subdomains in a regulated manner.

Complementing IFT, the BBSome, a conserved octameric complex, specializes in the trafficking of ciliary membrane proteins, particularly GPCRs [79]. Although loss of BBSome components can impair the ciliary localization of certain receptors [115, 116], it is now clear that its primary role lies in mediating the retrieval of activated GPCRs from cilia [117]. Upon activation, receptors such as SSTR3 and GPR161 are incorporated into large, processive retrograde BBSome trains in an Arl6-dependent manner and transported toward the ciliary base [118]. These receptors are often tagged with ubiquitin chains and recognized by β-arrestin, facilitating their removal from the cilium [119, 120]. When BBSome or β-arrestin function is compromised, activated GPCRs fail to exit the cilium efficiently and are instead shed via ectocytosis [121]. Thus, the BBSome is essential for refreshing ciliary signaling states by clearing activated receptors, functioning as a signal termination and baseline reset mechanism (Fig. 1C) that preserves dynamic responsiveness and prevents prolonged or inappropriate signaling.

At the base of the cilium, the TZ serves as a selective gating structure that maintains the molecular identity of the ciliary compartment. Structurally characterized by Y-shaped linkers connecting the axonemal microtubules to the ciliary membrane, the TZ is organized into distinct functional modules [80]. The MKS module forms a membrane-associated diffusion barrier, the NPHP module provides a structural scaffold on the axonemal side, and the CEP290 module acts as a central architectural hub linking these components [80]. Several mechanisms have been proposed for TZ function, including the formation of a sieve-like barrier that restricts passive diffusion of soluble proteins and regulates the entry of membrane-associated cargo [80]. Disruption of TZ components leads to profound defects in ciliary composition. For instance, ablation of TCTN1, a component of the MKS module, impairs the ciliary localization of proteins such as ARL13B, AC3, SMO, and TRPP2, and a human *TCTN1* mutation was determined to cause JS [122]. Joubert-syndrome and related disorders (JSRD)-associated mutations in CEP290 reduces the ciliary localization of key signaling components, including AC3 and ARL13B, yet paradoxically enhances HH signaling due to increased GPR161 exit, accelerated SMO entry, and elevated GLI2 accumulation at the ciliary tip [123]. In addition, AHI1, another MKS module component, facilitates the recruitment of ARL13B and 5HT6 to cilia, and its loss leads to altered axoneme structure and increased IFT-B [124]. These findings highlight that the TZ does not simply act as a passive barrier, but actively regulates the selective entry, retention, and distribution of signaling molecules within cilia. Functionally, the TZ can be viewed as implementing a selective filtering and boundary enforcement mechanism (Fig. 1D**)**, ensuring that only appropriate components are present while preventing contamination from the surrounding cellular environment.

Together, these trafficking and gating systems establish a highly regulated spatiotemporal framework that governs the availability and localization of signaling molecules within the primary cilium. By controlling the composition and dynamics of ciliary signaling components, they enable operations analogous to selective filtering (TZ), and baseline resetting (IFT/BBSome). Crucially, these processes provide the physical basis for the molecular computations described in Sect. "The modular organization of primary cilia signaling enables versatile molecular computation". Disruption of trafficking or gating does not merely impair signaling, but alters the computational logic of the cilium, leading to inappropriate signal persistence, reduced sensitivity, or increased noise.

### Structural plasticity of primary cilia endows versatility to signaling architecture

Primary cilia are not static organelles; rather, their structure is dynamically regulated in response to both extrinsic environmental cues and intrinsic cellular states. Changes in ciliary morphology and integrity provide an additional layer of control over signaling, complementing the modular signaling architecture and trafficking mechanisms described above. By altering the physical dimensions and composition of the cilium, structural plasticity is expected to influence the capacity and responsiveness of ciliary signaling, potentially serving as an adaptive mechanism to tune signaling output.

Ciliary length is one of the most prominent and regulated features of structural plasticity [81]. Ciliary GPCRs have been demonstrated to bidirectionally modulate cilia length. For instance, activation [125] or overexpression [126, 127] of the Gs-coupled 5HT6 promotes ciliary elongation, whereas activation of the Gi-coupled MCHR1 [128] leads to ciliary shortening. The inverse effects of 5HT6 and MCHR1 on cilia length could be partly attributed to their corresponding effects on cAMP levels, as direct manipulation of cellular cAMP has been demonstrated to regulate cilia length [129]. Importantly, such activity-dependent changes in cilia length suggest the existence of feedback mechanisms in which signaling inputs dynamically regulate ciliary architecture, which in turn modulates subsequent signaling responses. Structural plasticity of primary cilia also occurs in a temporally regulated manner. In the suprachiasmatic nucleus, neuronal cilia undergo rhythmic cycles of assembly and disassembly that are synchronized with circadian rhythms [130]. These structural oscillations have been proposed to modulate HH signaling and clock gene expression, thereby contributing to the coordination of neuronal activity underlying circadian behavior [130]. Although the molecular mechanisms governing these dynamics remain incompletely understood, such rhythmic remodeling suggests that cilia can impose a time-dependent modulation of signaling capacity. More broadly, given that circadian gene expression is widespread across the brain [131], similar ciliary dynamics may act as an overarching temporal filter (Fig. 1E**)**, prioritizing specific signaling responses according to time-of-day-dependent physiological demands.

Several molecular pathways have been implicated in the regulation of ciliary length. Aurora A kinase (AURKA) promotes ciliary disassembly through activation of histone deacetylase HDAC6, which destabilizes the axonemal microtubules [132]. Ciliary length is also inversely correlated with actin stress fiber formation in the cytoplasm [133]; increased actin polymerization may also drive the nuclear translocation of YAP/TAZ transcriptional regulators, which in turn promote the expression of AURKA and other negative modifiers of ciliogenesis [133–135]. Conversely, mTORC1 signaling promotes ciliary growth by enhancing the synthesis of ciliary proteins [136, 137]. Moreover, primary cilia are closely linked to autophagy: ciliary signaling can promote autophagosome formation, while autophagy regulates ciliogenesis and cilia length through the turnover of ciliary components [138–141]. Together, these pathways highlight the integration of cytoskeletal, metabolic, and proteostatic mechanisms in controlling ciliary architecture.

Despite these advances, the direct relationship between ciliary length and signaling output remains incompletely understood. Perturbations that alter cilia length often simultaneously affect protein trafficking or receptor localization, complicating interpretation. As discussed by Macarelli et al. [81], a key unresolved question is how the rate of protein trafficking scales with ciliary length. If trafficking rates scale proportionally, changes in length may preserve the density of signaling components, resulting primarily in changes in total signal capacity. In this framework, ciliary length may act as a form of variable gain control, tuning the overall magnitude of signaling output without necessarily altering its qualitative properties. In contrast, if trafficking fails to keep pace with structural changes, alterations in ciliary length could lead to changes in local concentration and interaction probability of signaling molecules, modulating signaling strength in a nonlinear manner. Addressing these possibilities will require quantitative approaches, such as single-molecule imaging [142, 143], to resolve the dynamics of protein distribution within cilia of varying lengths.

Besides gradual remodeling, primary cilia can undergo rapid structural changes. One such process is “cilia decapitation” in which the distal tip of the cilium is acutely shed [91]. This actin-dependent process is triggered during cell cycle re-entry and is associated with the removal of signaling components from the cilium. Mechanistically, the exit of INPP5E from ciliary membrane during G0-G1 transition leads to an accumulation of PI(4,5)P_2_ in the ciliary membrane, which recruits actin regulators that drive localized polymerization and membrane excision [91]. This process is enhanced in the absence of INPP5E, as observed in JS-associated contexts [91]. Notably, excised ciliary vesicles contain HH pathway components such as GLI3 and SUFU, and inhibition of growth-induced cilia decapitation reduces GLI activation, implicating a functional role in modulating signaling output [91]. A similar process has also been proposed as a mechanism for removing activated GPCRs. Whereas some receptors, such as SSTR3 and GPR161, are retrieved through BBSome-dependent trafficking, others e.g. NPY2R may be eliminated via extrusion from ciliary tips as extracellular vesicles [121]. Moreover, in BBSome or β-arrestin mutants where retrieval pathways are impaired, GPCRs may be diverted toward extracellular release [121]. Ciliary CDC42 has been implicated in regulating actin dynamics during this process [144]. Via the acute extracellular release of signaling components, cilia decapitation may function as a rapid bypass, or “short-circuit” mechanism that circumvents slower intracellular retrieval pathways (Fig. 1F), although its physiological relevance remains to be fully established.

Taken together, these findings demonstrate that structural plasticity introduces an additional layer of regulation that operates alongside signaling modules and trafficking mechanisms. By dynamically adjusting ciliary length and integrity, cells can modulate the effective concentration, distribution, and lifetime of signaling components within the cilium. In this way, structural plasticity enables operations analogous to variable gain, temporal filtering, and a circuit bypass mechanism (Fig. 1), allowing ciliary signaling to be tuned across different physiological contexts. When integrated with the molecular and trafficking frameworks described in Sects. "The modular organization of primary cilia signaling enables versatile molecular computation" and "Ciliary trafficking and gating enable molecular computation through spatiotemporal organization", this structural adaptability further expands the computational repertoire of primary cilia.

Across these examples, a unifying principle emerges: primary cilia are not passive conduits for signal transduction, but structured biochemical systems that transform inputs into context-dependent outputs through coordinated molecular operations. The modular organization of signaling pathways enables integration, amplification, and nonlinear transformation of inputs. Trafficking and gating mechanisms define the spatial and temporal availability of signaling components, implementing selective filtering and baseline reset mechanisms. Structural plasticity further modulates these processes by dynamically adjusting the physical dimensions and composition of the ciliary compartment, enabling primary cilia to actively reconfigure their architecture to optimize information processing in response to changing intrinsic and extrinsic conditions. Together, these features satisfy key criteria of molecular computation: the ability to integrate multiple inputs, to generate state-dependent outputs, and to dynamically adapt processing rules through changes in molecular composition and organization. In this view, primary cilia function as specialized computational microdomains within cells [41], where signaling is not merely relayed but actively processed to shape downstream transcriptional, physiological, and behavioral outcomes.

## Primary cilia shape neural computation through brain development

The computational functions of primary cilia described in Sect. "Primary cilia as molecular computational microdomains" extend beyond single-cell signaling to influence the development of neural circuits. During brain development, primary cilia integrate extracellular cues and intrinsic cellular states to regulate key processes such as neurogenesis, cell fate specification, and neuronal migration. Through these roles, ciliary signaling shapes the number, identity, and spatial organization of neurons, thereby establishing the structural and functional architecture upon which neural computation depends. The following subsections examine how ciliary molecular computation during development scales to influence circuit-level information processing.

### Regulation of neurogenesis and neuronal number

In the examples below, we discuss how ciliary signaling influences proliferative self-renewal and differentiation decisions in neural progenitor populations.

In the developing cortex, the proliferation of RG progenitors is repressed by primary cilia. Cilia disassembly induced by IGF-1 and LPA or cilia instability induced by INPP5E mutations bias cortical RG progenitors towards cell cycling and away from neuronal differentiation [85, 145–147]. Conversely, cilia elongation induced by LPA receptor LPAR1 deletion decreased RG proliferation [145]. Furthermore, inhibited ciliogenesis resulting from a CROCCP2 gain-of-function mutation has also been shown to enhance cortical progenitor amplification [148]. Interestingly, signal transduction from ciliary GPR157 has been shown to promote RG neuronal differentiation [149].

This function for the primary cilium may differ across neuroanatomical regions: in the cerebellum, the primary cilium mediates mitogenic SHH signaling that promotes proliferative outcomes. Formation of the primary cilium in cerebellar GNPs is mediated by the ATOH1 transcription factor which promotes GNP proliferation, whereas the E3 ubiquitin ligase HUWE1 promotes GNP differentiation via cilia disassembly. Owing to their functions in maintaining ciliary structure and the proliferation-differentiation balance, the over-expression of ATOH1 and the under-expression of HUWE1 have been linked to the formation of pediatric brain tumorigenesis [150, 151].

The correlation between mitogenic ciliary HH signaling and proliferative outcomes observed in cerebellar neural progenitors has also been observed in NSCs of the postnatal hippocampus. Inhibition of cilia assembly or HH-associated SMO resulted in hippocampal hypoplasticity due to deficiency of astrocyte-like neural precursors or radial astrocytes in the dentate gyrus [152–154].

Regulating neurogenesis can have a significant effect on the final numbers of neurons within the developed brain, affecting cognitive functions and behavior. Through complex interactions at the primary cilium, the proliferation-differentiation balance of these neural progenitors is tightly controlled to maintain a pool of proliferative cells while producing neurons through differentiation. Ultimately, it is clear that dysfunction of ciliary processes can result in erroneous neuroanatomical development, such as altered neuronal density in the newborn cortex [145, 146] or hypoplastic hippocampal regions in perinatal mice [146, 153].

### Control of cell fate specification

Primary cilia are also involved in the specification of neuron subtype produced through differentiation. In the ventral spinal cord, ciliary SHH signaling via PTCH1 is modulated by NOTCH signaling, contributing to dorsoventral patterning and the specification of neural progenitor fates [155]. Loss of TZ protein FTM/RPGRIP1L eliminates functional cilia in telencephalic progenitors, disrupting cell fate specialization in the forebrain and producing dorsalization defects such as missing telencephalic structures in *FTM*-/- embryos [156, 157]. Investigation into human embryonic stem cell (hESC) differentiation further suggest that primary cilia can indirectly repress the expression of pluripotency factors OCT4 and NANOG, committing hESCs towards a neuroectoderm fate and abandoning the alternative mesendoderm fate [158].

### Regulation of neuronal migration and positioning

Radial glia (RG) serve as both neural progenitors and a structural scaffold that guides neuronal migration, thereby ensuring the proper spatial organization of neurons during brain development. The ability of RG to fulfill these roles depends critically on their apico-basal polarity, which is established and maintained by primary cilia and associated signaling pathways. Disruption of ciliary structure or function impairs RG polarity and consequently affects both neurogenesis and neuronal positioning. In early postnatal mice, loss of TALPID3, a ciliary base protein, disrupts RG scaffolding and progenitor localization in the dentate gyrus [154]. Similarly, mutations in ciliary genes such as ARL13B, BBS1, BBS7, BBS10, AHI1, and ALMS1 impair the establishment of polarity in RG and newborn neurons, leading to defects in radial migration [159, 160].

Beyond their role in RG organization, primary cilia also regulate the migratory behavior of neurons. Cortical interneurons originating from the medial ganglionic eminence rely on cilia-mediated SHH signaling to transition from tangential to radial migration and enter the cortical plate [161]. In addition, ciliary signaling influences neuroblast migration more broadly, contributing to the precise positioning of neurons within developing circuits [162].

Notably, neuronal positioning is further refined during postnatal development through reverse migration, in which neurons adjust their positions after initial radial movement. The orientation and integrity of primary cilia have been implicated in this process, as disruption of ciliary proteins such as IFT88 and ARL13B impairs reverse migration and alters cortical lamination [163].

In the examples above, the lack of a primary cilium had significant impact on neuronal development [154, 159, 160, 162], suggesting an important organellular output from ciliary signal processing that shapes transcriptomic profiles in the developing neuron [164, 165]. Moreover, the region-specific associations between primary cilia persistence and neurogenesis point towards more complex state-dependent signaling, likely involving extracellular or intracellular input.

### Control of neurite development and circuit connectivity

Beyond regulating neuronal production and positioning, the establishment of functional neural circuits depends critically on the growth, patterning, and targeting of neurites. Emerging evidence suggests that primary cilia contribute to these processes by coordinating signaling pathways that regulate dendritic development, axonal guidance, and synaptic connectivity.

In developing cortical neurons, ciliary signaling has been implicated in the regulation of dendritic growth. Disruption of ARL13B alters ciliary calcium signaling, while overexpression of the ciliary GPCR 5HT6 depletes adenylate cyclase 3 (AC3) from primary cilia [127, 164]. These perturbations are associated with reduced dendritic outgrowth in both pyramidal neurons and inhibitory interneurons, and can be rescued by restoring ciliary signaling through AC3 or SSTR3 [127, 164], highlighting the importance of ciliary cAMP- and calcium-dependent pathways in dendritic development.

Primary cilia also regulate axonal guidance through both cilia-dependent and cilia-independent mechanisms. While ARL13B has been reported to function within growth cones independently of cilia [166], subsequent studies demonstrate that ARL13B and IFT88 mediate commissural axon guidance via ciliary SHH signaling through SMO [35]. In this context, proper ciliary function is required for transcriptional regulation of Hedgehog-interacting protein (HHIP), which contributes to accurate axon turning [35].

In addition to long-range guidance, ciliary signaling influences axonal tract formation and connectivity. In deep cerebellar neurons, ARL13B regulates axonal growth and orientation, likely through downstream INPP5E and PI3K-AKT signaling pathways [165]. Loss of ARL13B leads to misoriented axonal tracts and altered growth cone dynamics, while targeted manipulation of ciliary calcium, cAMP, and phosphoinositide signaling rapidly modulates growth cone behavior in cultured neurons [165]. Consistent with these findings, disruption of other ciliary regulators, such as the transcription factor RFX3 or INPP5E, results in aberrant thalamocortical projections, including misrouting of axons to inappropriate targets such as the amygdala [167].

Finally, ciliary dysfunction can impact synaptic development. In interneurons, deletion of ARL13B alters axonal bouton structure and disrupts synapse formation [164], suggesting that ciliary signaling extends beyond neurite outgrowth to influence the establishment of functional connectivity.

Together, these findings indicate that primary cilia act as signaling hubs that integrate second messenger pathways to regulate multiple stages of neurite development, from dendritic growth and axon guidance to synaptic connectivity, thereby linking subcellular signaling dynamics to the assembly of functional neural circuits.

### From development to circuit computation: roles of primary cilia

The developmental roles of primary cilia described in Sects. "Regulation of neurogenesis and neuronal number"–"Control of neurite development and circuit connectivity" shape the structural architecture from which neural computation emerges. By regulating neurogenesis, cell fate specification, neuronal migration, and neurite development, primary cilia influence neuronal number, identity, positioning, and connectivity. In this way, ciliary molecular computation during development is translated into circuit-level computation by defining the cellular units and wiring logic that support information processing.

Developmental disruption of ciliary signaling can therefore compromise neural computation at multiple levels. Altered neurogenesis may change neuronal density, as seen in hippocampal hypoplasia [146, 153], potentially impairing learning processes that require sufficient neuronal populations [168]. Defective migration can produce heterotopias or polymicrogyria, which have been reported in patients with JS [169] and are associated with epilepsy, intellectual disability, and SCZ [169–171]. Such abnormalities disturb the spatial organization of brain networks, whose efficient three-dimensional topology, dense signaling hubs, and modular organization are increasingly recognized as central to brain computation [172–175]. By controlling proliferation and migration, primary cilia therefore contribute to the construction of computationally efficient circuit architecture.

Primary cilia also influence communication fidelity within and between circuits. Local computation depends on interneuron-mediated E/I balance [176], and ciliary disruption of interneuron neurite development can impair inhibitory connectivity [164], potentially causing network hyperexcitability and reduced signal fidelity. Long-range computation depends on axonal projections, including commissural pathways that support interhemispheric information transfer and decision-making [177]. Because guidance of these projections requires intact ciliary signaling [35], cilia may also contribute to the integration of distributed neural activity.

Together, these findings suggest that primary cilia support neural computation not only through acute signaling in mature neurons, but also by shaping the developmental processes that establish circuit architecture. By controlling neuronal number, identity, positioning, and connectivity in a context-dependent manner, ciliary signaling links subcellular molecular computation to systems-level information processing.

## How ciliary GPCRs influence computational processes in the adult brain

In Sect. "Primary cilia shape neural computation through brain development", we discussed how primary cilia play pivotal roles in orchestrating embryonic and early postnatal brain development. Primary cilia in the brain persist into adulthood, where they are well positioned to continue influencing cellular and circuit-level processes. While their developmental roles suggest a continued contribution to adult neurogenesis [152, 178], an equally important and less explored aspect is their presence in most mature neurons across the adult brain.

Notably, neuronal primary cilia are enriched with a diverse array of neuromodulatory GPCRs. In the striatum, receptors such as 5HT6 [179], DRD1 [180], and MCHR1 [181] are selectively localized to neuronal cilia, whereas in the hypothalamus, MC4R [182] and NPY2R [183] are enriched in neuronal cilia within the paraventricular and arcuate nuclei, respectively. Although these GPCRs are well known to regulate neuronal activity and behavior, it remains unclear how their compartmentalization within primary cilia contributes to their functional specificity.

In the following sections, we examine emerging evidence that ciliary GPCRs can modulate neuronal properties at both the single-cell and circuit levels. By highlighting these examples, we aim to illustrate how primary cilia provide a specialized subcellular platform through which neuromodulatory signals are selectively processed, ultimately shaping the balance and dynamics of neural activity in the adult brain.

### Ciliary 5HT6 regulates properties of axon initial segment

5HT6 is a serotonin receptor enriched in neuronal cilia of various brain regions involved in memory and learning, such as the cerebral cortex, striatum, hippocampus, cerebellum, and olfactory tubercle [179, 184–186]. Multiple 5HT6 antagonists have made it to clinical trials as treatments for the cognitive effects of neurodegenerative or psychiatric diseases [187–190], some of which have found moderate improvements in cognitive performance [187, 188]. In rodent models, 5HT6 antagonists have produced cognitive enhancement in tasks such as spatial learning [125, 191, 192], memory retention [191–193], and object recognition [194]. In contrast, agonism of the 5HT6 receptor has produced conflicting results, with evidence supporting pro-cognitive effects [195, 196] as well as impaired cognition [197, 198]. Given that 5HT6 exhibits basal constitutive activity, antagonists are likely to suppress ongoing physiological signaling, whereas agonists may drive supraphysiological activation. This distinction may contribute to conflicting experimental outcomes, depending on the brain regions and circuits engaged. At the systems level, 5HT6 enables autoregulation of serotonin release by mediating serotonergic neuron activity [199], and regulates the neurotransmission of acetylcholine [200, 201], glutamate [202–204], and catecholamines [205, 206].

More recently, the 5HT6 signaling pathway has been shown to modulate the length of neuronal cilia, as well as structural characteristics of the AIS, suggesting a functional connection between these two neuronal sub-compartments. Overexpression of 5HT6 in cultured [125, 207] and in vivo [185] neurons increased primary cilia length, elongated the AIS [125, 207], and shifted the AIS more proximally towards the soma [125]. Blockade of this pathway either via genetic or pharmacological means produced the opposite effects in cultured neurons [125, 185, 207]. Positive correlations between neuronal cilia and AIS length have also been observed when other ciliary proteins, including IFT88 and SSTR3, were overexpressed [207, 208], suggesting convergence onto shared downstream mechanisms. In cultured mouse hippocampal neurons, 5HT6 overexpression was associated with reduced intrinsic excitability, including decreased action potential amplitude and firing frequency [207]. Moreover, in an APP/PS1 Alzheimer’s mouse model, 5HT6 expression and neuronal cilia length commonly increased in the hippocampus, and these were similarly associated with a longer AIS and shorter AIS-soma distance [125]. By applying a 5HT6 antagonist, these ciliary and AIS phenotypes reversed, and improvement was observed in spatial memory [125]. Together, these studies suggest that ciliary 5HT6 signaling may regulate physical properties of the AIS, modulating the integration of excitatory and inhibitory postsynaptic potentials (EPSPs and IPSPs) to control neuron firing, thereby influencing computation at a single-neuron level.

**Fig. 2:**
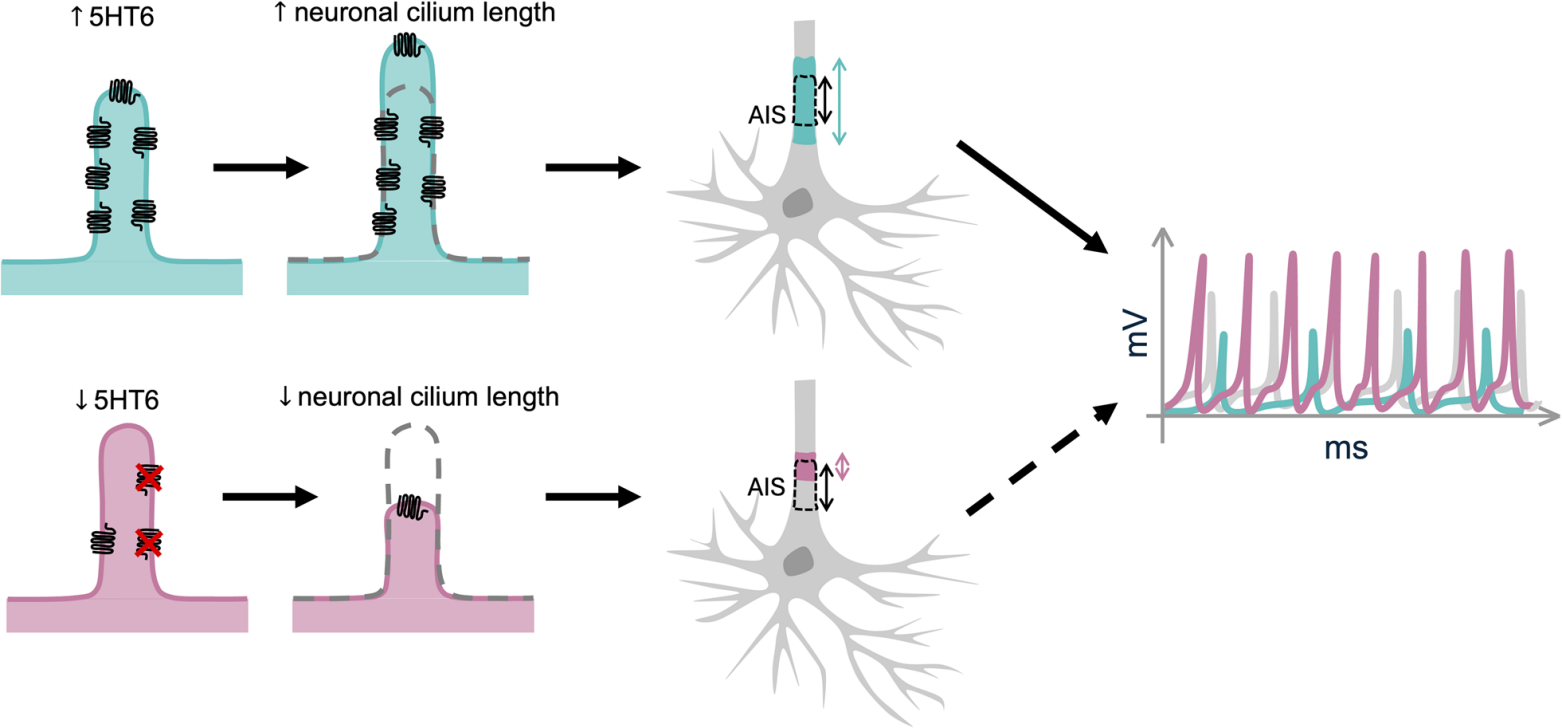
Ciliary 5HT6 regulates neuronal excitability through modulation of axon initial segment (AIS) structure and positioning. In the hippocampus, changes in 5HT6 receptor abundance alter primary cilium length and are associated with corresponding shifts in AIS morphology and localization. Increased 5HT6 expression elongates primary cilia and correlates with an elongated AIS (blue) positioned more proximally to the soma, resulting in reduced neuronal excitability, as reflected by decreased frequency and amplitude of excitatory postsynaptic currents (EPSCs) relative to baseline (gray) [207]. Conversely, reduced 5HT6 shortens primary cilia and is associated with a shortened AIS (pink) that is displaced distally from the soma [125]. The functional consequences of this shortened AIS remain to be fully resolved, although it may plausibly increase neuronal excitability

Figure 2 illustrates this proposed mechanism, showing how ciliary 5HT6 signaling modulates the structural relationship between primary cilia and AIS in hippocampal neurons. While the functional mechanism linking primary cilia and the AIS remain to be fully elucidated, a recent study suggests that loss of 5HT6 in the mouse hippocampus is associated with reduced Ankyrin-G expression, potentially mediated by decreased CREB-dependent transcription at the AnkG promoter [208]. The relationship between 5HT6 signaling and neuronal excitability may represent a form of negative feedback within hippocampal circuits. Ciliary 5HT6 activity could dampen neuronal excitability to correct for the over-stimulation, whereas mechanisms that perturb 5HT6 signaling or ciliary structure may promote neuronal excitability.

Although a positive correlation between primary cilia length and AIS length has been observed across several contexts [125, 207, 208], the relationship between AIS structure and neuronal excitability is complex. In particular, the impact of AIS length must be considered alongside the neuron type [209], the position of AIS relative to the soma, as well as the distribution and density of ion channels within the AIS [210]. Computational models predict that small neurons are most excitable when the AIS is proximally located and of intermediate length, whereas neurons with larger dendritic arbors achieve maximal excitability with a longer and more distally positioned AIS [50]. Therefore, systematic studies examining how ciliary GPCR signaling influences AIS architecture and neuronal excitability across different cell types will be essential for understanding how these two subcellular domains interact to shape input integration at the single-neuron level. Through the reshaping of AIS, neurons are able to dynamically adjust their threshold for depolarization, allowing for an additional tuneable parameter to impact the fundamental computation of neurons of analogue input to digital output in response to shifting network requirements.

### Ciliary SSTR3 modulates excitation-inhibition (E/I) balance in neural circuits

SSTR3 is a ciliary GPCR for somatostatin that has been implicated in spatial and object recognition memory [208, 211]. Ciliary SSTR3 signaling has been implicated in the regulation of synaptic properties. In cultured rat pyramidal neurons, acute knockdown of ciliary proteins, including ARL13B, IFT88, and CEP164, upregulated the density of excitatory synapses and the accumulation of postsynaptic AMPA receptors, without affecting inhibitory synapses [212]. These changes were accompanied by enhanced AMPAR-mediated glutamatergic currents and increased spontaneous neuronal firing. Pharmacological manipulation further suggested that these effects are mediated, at least in part, by ciliary SSTR3, as antagonism increased excitatory synaptic strength whereas agonism produced the opposite effect [212]. Notably, these findings contrast with observations from SSTR3 knockout mice, in which hippocampal neurons exhibited reduced excitatory spine density and decreased spontaneous activity [208]. This apparent discrepancy likely reflects differences in experimental context, including acute versus chronic perturbation, cell-autonomous versus circuit-level effects, and potential compensatory mechanisms in the knockout model. Beyond pyramidal neurons, ciliary signaling also exerts non-cell-autonomous effects. Developmental loss of ARL13B in mouse interneurons impaired axonal and dendritic growth, leading to reduced synaptic connectivity with surrounding medium spiny neurons in the striatum and a consequent shift in the E/I balance [164]. Importantly, these circuit-level deficits could be rescued by restoring SSTR3 expression in the mutant interneurons [164], highlighting a role for ciliary SSTR3 signaling in maintaining network connectivity and synaptic balance.

The E/I balance of neural circuits reflects a dynamic interplay between signal propagation and suppression across multiple scales [213]. At the subcellular level, ciliary signaling contributes to this balance through opposing Gs and Gi pathways that regulate local second-messenger dynamics [82]. At the single-neuron scale, opponent excitatory and inhibitory conductance are spatially distributed across the somatodendritic arbor before integration at the AIS [213]. At the circuit scale, this balance is maintained through the integration of excitatory and inhibitory inputs and the coordinated activity of interneuron networks, stabilizing neural circuits against entering states of sustained hyper- or hypo-activity [213]. Disruption of these mechanisms leads to altered E/I ratios, which are associated with neurological conditions including SCZ [214], epilepsy [75], and ASD [71, 215, 216].

**Fig. 3:**
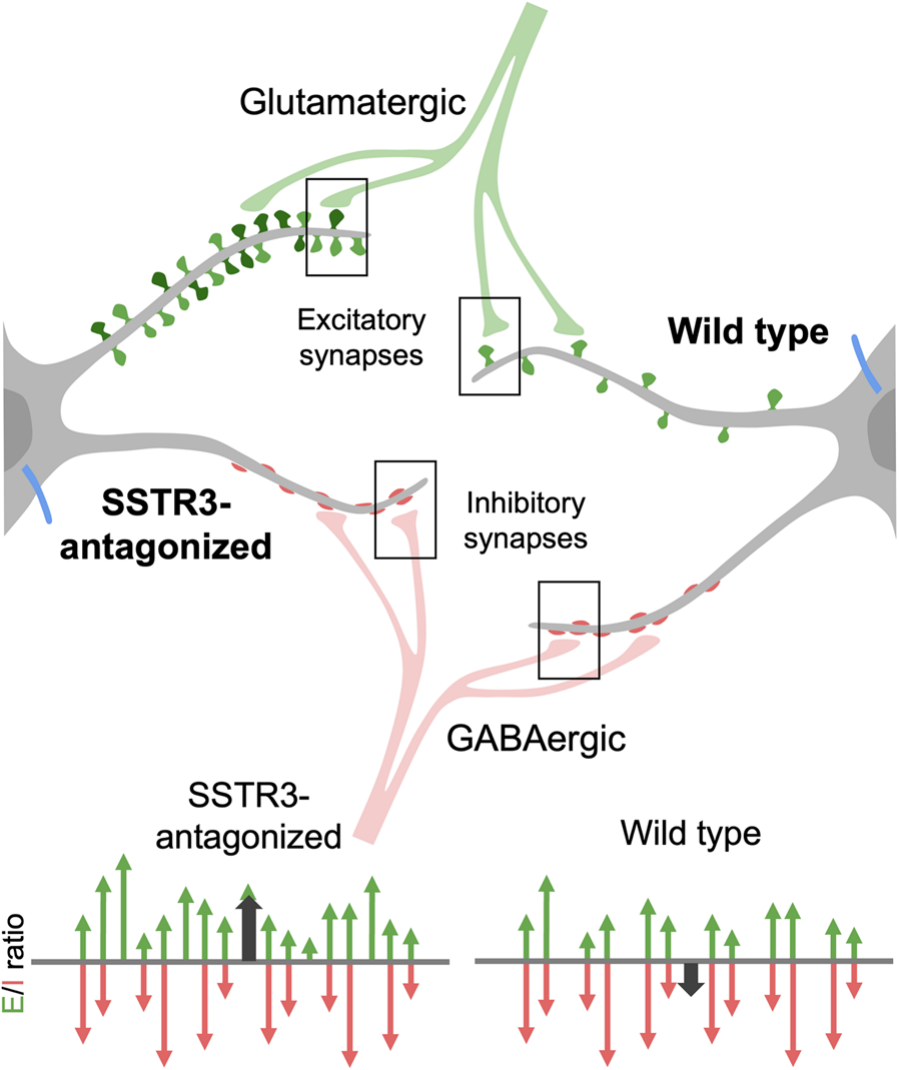
Ciliary SSTR3 modulates E/I balance across cellular and population scales. SSTR3 antagonism in cultured pyramidal neurons selectively enhances excitatory synaptic strength and density, without altering inhibitory synapses, indicating that ciliary SSTR3 constrains excitatory synapse formation and/or maturation [212]. This selective regulation shifts the excitatory-inhibitory (E/I) balance at the level of individual neurons toward increased excitation. At the population level, cumulative shifts in single-neuron E/I balance propagate to alter overall network activity. In the schematic below, individual neurons are represented as excitatory (upward arrows) or inhibitory (downward arrows) units, illustrating how cell-autonomous changes in synaptic drive scale to population-level effects. The net circuit E/I state is summarized by the thick black arrow

In this context, ciliary SSTR3 signaling may contribute to the neuromodulatory tuning required to maintain circuit stability. As illustrated in Fig. 3, a neuron population may exhibit a net inhibitory tone at rest under normal conditions. Upon SSTR3 inhibition, however, enhanced excitatory drive may override this inhibitory tone, leading to aberrant activation of neural pathways. These findings suggest that regulation of E/I balance may originate, in part, from subcellular ciliary signaling mechanisms, with downstream consequences for circuit function and behavior [211], as discussed further in Sect. "An emerging role of primary cilia and brain memory".

### The rheostat function of ciliary MC4R in antagonistic feeding neural circuits

Physiological states such as hunger and satiety are not binary, but instead emerge gradually and must be maintained over extended timescales ranging from minutes to hours or longer [60]. This presents a fundamental challenge for neural circuits, as fast neurotransmitter synaptic transmission operates on millisecond timescales and is therefore insufficient to sustain stable motivational states [217]. We propose that neuromodulatory GPCRs enriched in primary cilia, such as MC4R, function as rheostat-like regulators that integrate signals over time to generate slow, graded outputs. In this framework, ciliary GPCR signaling does not simply switch neuronal activity on or off but instead provides a continuous modulatory influence that stabilizes circuit output and maintains behavioral states.

**Fig. 4:**
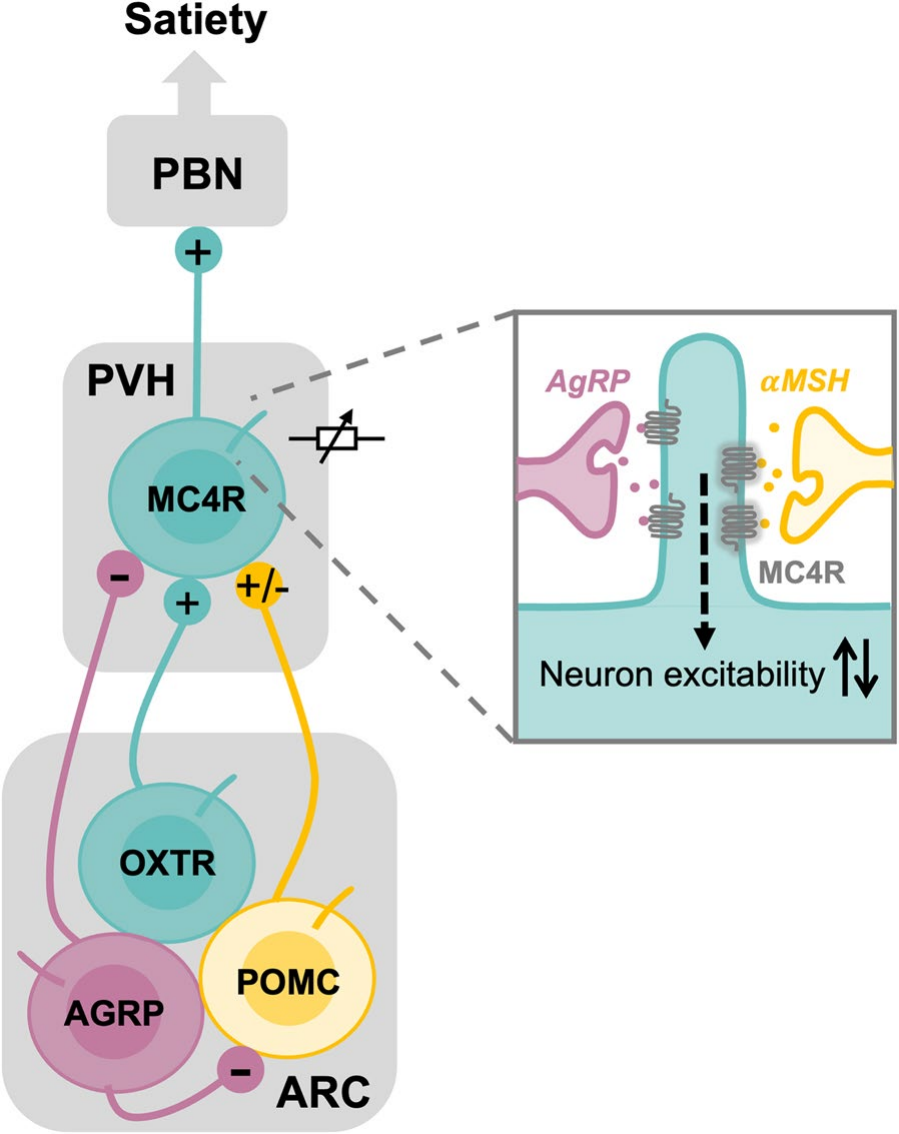
Primary cilia function as neuromodulatory rheostats within antagonistic feeding circuits. MC4R-expressing neurons in the paraventricular hypothalamus (PVH) project to the parabrachial nucleus (PBN), forming a core satiety-promoting circuit. The primary cilium of PVH MC4R neurons integrates opposing neuromodulatory inputs from arcuate nucleus (ARC) populations. POMC neurons release α-MSH to activate MC4R, increasing PVH neuronal excitability, whereas AgRP neurons release AgRP, an inverse agonist of MC4R, suppressing receptor signaling and reducing neuronal activity. Through this antagonistic signaling, ciliary MC4R functions as a bidirectional rheostat that computes competing inputs to tune PVH excitability and satiety output. In parallel, PVH MC4R neurons receive excitatory inputs from ARC OXTR-expressing neurons, providing an additional layer of modulation. Together, these mechanisms position ciliary MC4R as a node-specific regulator that dynamically adjusts the strength of satiety signaling within the ARC-PVH-PBN circuit

Within the hypothalamus, feeding behavior is regulated by antagonistic neural circuits linking arcuate nucleus (ARC) neurons to MC4R-expressing neurons in the paraventricular hypothalamus (PVH) [61, 62] (Fig. 4). PVH-MC4R neurons encode satiety by projecting to downstream brainstem regions that suppress appetite [218–221]. These neurons receive opposing inputs from upstream ARC populations: excitatory oxytocin receptor (OXTR)-expressing neurons promote PVH-MC4R activity via glutamatergic transmission [219, 222], whereas AGRP neurons suppress their activity through GABAergic signaling [219, 223]. Moreover, POMC neurons are heterogeneous and can release either glutamate or GABA, mounting rapid excitatory or inhibitory responses on PVH-MC4R neurons [62, 224, 225]. Such rapid neurotransmitter-based communication between ARC and PVH functions as fast on–off switches of feeding behavior.

Beyond fast synaptic transmission, ARC neurons also modulate PVH-MC4R activity through slower neuromodulatory signaling (Fig. 4). MC4R, one of the strongest genetic determinants of human obesity [226–228], is a Gs-coupled GPCR that raises intracellular cAMP levels and triggers downstream PKA signaling [63, 229]. ARC-POMC neurons release α-MSH, an MC4R agonist, to sustain excitability of PVH-MC4R neurons [63, 64]. In contrast, ARC-AGRP neurons secrete agouti-related peptide (AgRP), which acts both as a competitive antagonist of α-MSH at MC4R and as an endogenous inverse agonist to provide a prolonged inhibitory tone on PVH-MC4R neuron activity [65, 66]. Of note, several obesity-associated mutations in MC4R impair its ciliary localization [182]. Genetic ablation of primary cilia in PVH-MC4R neurons, or inhibition of ciliary AC3, disrupts MC4R-dependent satiety signaling and leads to hyperphagia and obesity [182, 230]. These findings demonstrate that proper ciliary localization is essential for MC4R-mediated control of feeding behavior. Elucidating downstream ciliary cAMP-dependent signaling pathways, including potential transcriptional mechanisms, will be key to understanding the basis of these slow, graded responses.

Nevertheless, these observations support a model in which ciliary MC4R functions as a rheostat within the core ARC-PVH feeding circuit, with receptor activity (and consequently PVH neuronal output) continuously tuned by competing neuromodulatory inputs rather than simply switched on or off. The relative levels of α-MSH and AgRP dynamically adjust MC4R signaling output, modulating cAMP-PKA activity over a graded range [63–66]. Because these ligands are released in a sustained and state-dependent manner, MC4R signaling integrates their cumulative influence over time, effectively encoding the balance between anorexigenic and orexigenic drives as a continuous variable. In this way, MC4R does not dictate discrete behavioral states, but instead regulates the intensity and persistence of feeding-related neural activity [222, 231]. Rather than producing rapid, binary outputs, bidirectional control of MC4R integrates neuromodulatory inputs over time to generate slow, graded signals that stabilize feeding states. This helps explain naturalistic feeding behavior: hunger builds progressively, driving food-seeking behavior, while satiety emerges gradually rather than abruptly terminating feeding [60].

Beyond these receptors, hypothalamic neuronal cilia also host additional metabolic GPCRs, including NPY2R [115] and MCHR1 [232], which convey signals related to energy balance, arousal, and food-seeking. By co-localizing multiple neuromodulatory receptors within a confined ciliary signaling compartment, hypothalamic neurons may integrate diverse interoceptive cues (nutrient status, hormone levels, metabolic peptides) with exteroceptive cues (environmental safety, food availability). Collectively, this arrangement allows primary cilia to act as hubs for state integration, supporting smooth transitions between hunger and satiety and enabling adaptive feeding behavior that matches internal needs with external opportunities. Importantly, in many ciliopathies, this rheostat-like modulation is disrupted. Without proper ciliary signaling, the system loses its ability to regulate feeding tone, often resulting in excessive, unregulated hyperphagia and obesity.

### Dynamic partitioning of DRD1 between cilia and dendrites shapes dopaminergic signaling

Among ciliary GPCRs, DRD1 represents a notable exception to the canonical model of stable ciliary enrichment. Most ciliary GPCRs, such as SSTR3 or GPR161, are highly enriched within the primary cilium at baseline and are gradually removed only upon activation [118, 119, 121]. In contrast, DRD1 exhibits a markedly different behavior. While overexpression studies demonstrate that DRD1 can localize to primary cilia in both non-neuronal cells [233] and neurons [234], endogenous DRD1 in the brain is predominantly detected in somatodendritic compartments, with only minimal steady-state enrichment in cilia [180, 234]. Strikingly, disruption of BBSome-mediated trafficking leads to robust accumulation of DRD1 within cilia without altering total receptor expression [180, 234]. These observations suggest that DRD1 is not stably resident within cilia but instead undergoes continuous trafficking in and out of the ciliary compartment, with its steady-state localization determined by the balance between entry and export. This is consistent with earlier studies depicting that D1 receptors undergo dynamic trafficking across neuronal compartments including dendritic spines, shafts, and the soma [235–238].

**Fig. 5:**
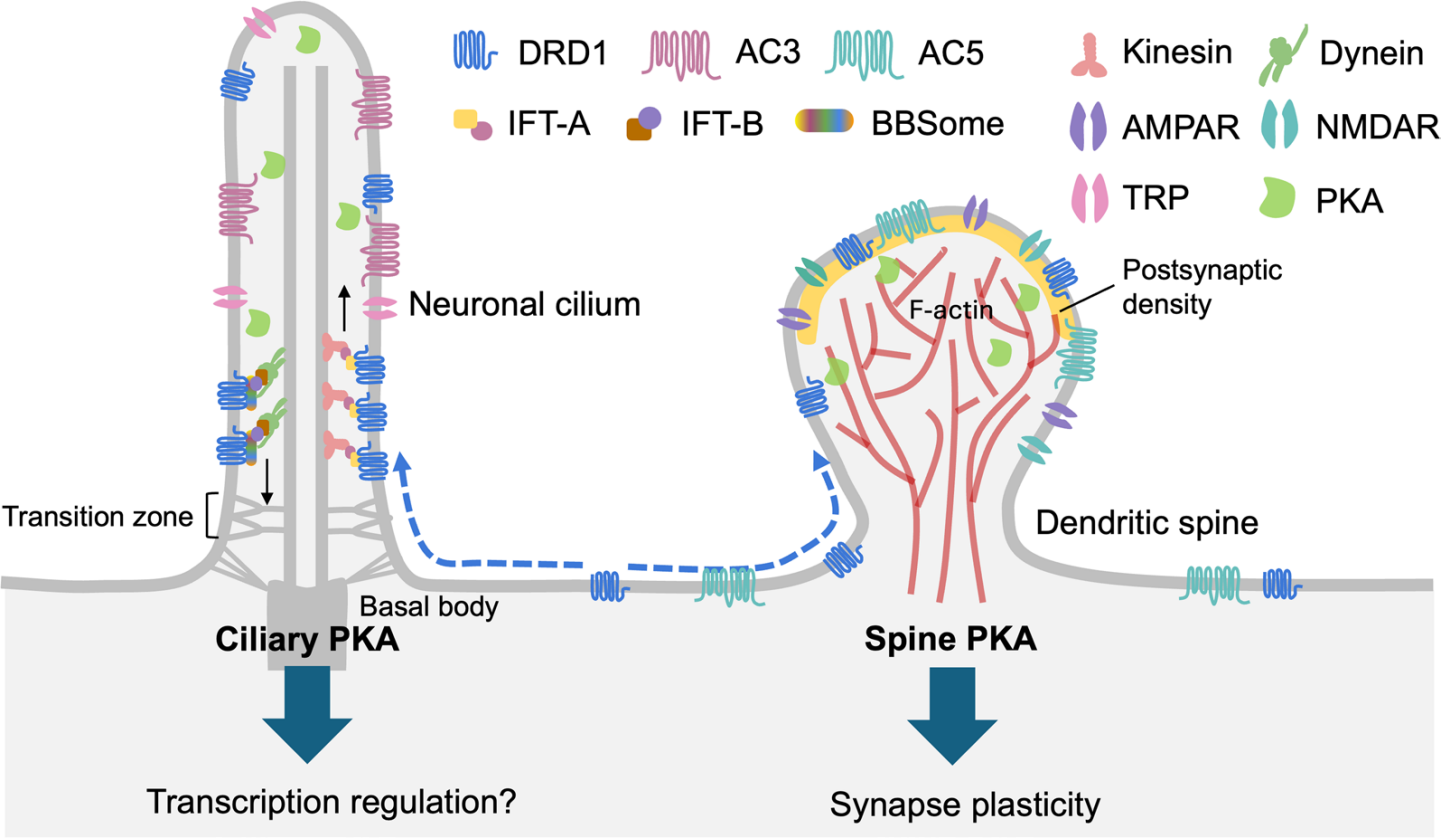
Compartmentalized DRD1 signaling in dendritic spines and primary cilia. In DRD1-expressing neurons, such as direct pathway medium spiny neurons of the striatum, DRD1 receptors are distributed across dendrites, soma, and the primary cilium. DRD1 localization is dynamic, with ciliary retention regulated by IFT/BBSome-mediated trafficking and the transition zone diffusion barrier. Distinct adenylate cyclase isoforms couple DRD1 to compartment-specific cAMP signaling, with AC3 enriched in the cilium and AC5 predominating in dendritic spines. In dendritic spines, DRD1-driven cAMP-PKA signaling regulates synaptic structure and function by modulating actin dynamics and AMPAR/NMDAR composition, thereby shaping long-term potentiation and depression (LTP/LTD). In contrast, the functional consequences of ciliary DRD1 signaling remain less well defined. Given that ciliary PKA has been shown to regulate CREB-dependent transcription in non-neuronal systems [246], we propose that ciliary DRD1 signaling may similarly engage CREB to modulate neuronal gene expression

In dendritic spines and shafts, DRD1 is well established to regulate synaptic plasticity through AC5-cAMP-PKA signaling. Activation of spine-localized DRD1 enhances PKA activity, which phosphorylates key synaptic substrates including AMPA and NMDA receptor subunits [239–242], and modulates actin cytoskeleton dynamics to influence spine morphology [243–245]. Through these mechanisms, DRD1 lowers the threshold for long-term potentiation (LTP) and contributes to activity-dependent synaptic strengthening [239–242]. Thus, somatodendritic DRD1 functions as a rapid and spatially localized modulator of synaptic efficacy. Given this established role, even in the absence of a defined ciliary signaling function, the cilium may directly influence computation at the single-neuron level by regulating the subcellular distribution of DRD1. Sequestration of DRD1 away from dendritic compartments and into the cilium would be expected to reduce its availability at synapses, thereby indirectly attenuating PKA-dependent modulation of synaptic plasticity (Fig. 5). Conversely, efficient retrieval of DRD1 from cilia via BBSome-mediated trafficking [180, 234] would maintain its somatodendritic pool and preserve its role in fast synaptic signaling. In this view, the primary cilium may act as a regulatory reservoir that tunes the strength of synaptic DRD1 signaling.

The accumulation of DRD1 within cilia observed in BBS mutant neurons raises an additional and intriguing possibility: that ciliary DRD1 may engage distinct signaling pathways when retained within this compartment. As a Gs-coupled receptor, DRD1 is expected to activate adenylate cyclases, and within cilia, this would likely involve AC3 [89] rather than the AC5 [244] isoforms predominant in somatodendritic membranes. Such compartmentalized cAMP production could activate a ciliary PKA pool [92, 108], potentially analogous to signaling mechanisms described in HH pathways, where PKA regulates downstream transcriptional programs [108]. One candidate effector is the transcription factor CREB, which has been detected in primary cilia in non-neuronal systems [246] and could, in principle, respond to ciliary PKA signaling and translocate to the nucleus where it may regulate the induction of immediate early genes (IEGs) such as *C-FOS*, *EGR1* [247, 248], as well as the delayed expression of downstream plasticity-related genes, including neurotrophic factors like BDNF [249], thereby potentially contributing to longer-term structural and functional neural adaptations. While this model remains speculative, it suggests that prolonged retention of DRD1 in cilia may shift dopaminergic signaling toward slower, transcriptionally mediated responses (Fig. 5).

Taken together, these observations support a model in which primary cilia regulate DRD1 function through two complementary mechanisms. Under physiological conditions, dynamic trafficking may allow cilia to act as a buffering compartment that controls the availability of DRD1 for synaptic signaling. Under pathological conditions, such as in BBSome dysfunction, accumulation of DRD1 within cilia may not only deplete its synaptic pool but also enable engagement of alternative ciliary signaling pathways. Thus, rather than functioning solely as a static signaling organelle, the primary cilium may serve as a dynamic regulator of receptor partitioning, shaping the balance between fast synaptic modulation and slower, integrative signaling modes.

## An emerging role of primary cilia and brain memory

We will now discuss the potential ciliary role in impacting the memory mechanisms facilitating neural computation. While classical views relegate memory to a passive storage mechanism [250, 251], strong convergent evidence has shifted the field’s perspective in realizing how the highly dynamic nature of memory formation and retrieval processes actively shape information processing by controlling input–output to the computational centers [252, 253]. Decision-making rarely occurs in a vacuum, giving rise to the concept of state-dependent computation [254, 255], in which the selection of an action depends not only on current inputs but also on hidden context and the history of previous outcomes [253]. Reflecting this principle, the best-performing frameworks for modeling adaptive behavior and complex human strategy rely on integrating past experiences to improve the predictability of future outcomes: Hidden Markov Models for behavioral state transitions [256], deep reinforcement learning architectures like AlphaGo [257], and memory-augmented Recurrent Neural Networks [258].

The fundamental physical unit of memory is the engram, a distributed ensemble of neurons whose stimulation leads to memory retrieval even in the absence of external or intrinsic signals [259]. Engram formation, reconsolidation, and extinction rely on two key properties: stability and competition [260, 261]. When the mechanisms underlying these properties fail, whether through an inability to stabilize transient traces, synchronize interregional networks, or separate overlapping inputs, the network's recall-based computational capacity will likely become disrupted. This can be assessed through distinct performance deficits across working memory (WM), associative learning, and spatial discrimination assays [259, 262]. Strikingly, targeted perturbations of the primary cilium have been empirically shown to elicit some of these behavioral failures [178, 211, 263, 264]. To understand how ciliary dysfunction may translate to these macroscopic deficits, the following sections will examine the primary cilium’s involvement in several brain plasticity mechanisms underlying memory, namely intracellular Synaptic Tagging and Capture (STC), intercellular Spike-Time Dependent Plasticity (STDP), and structural plasticity via Adult Hippocampal Neurogenesis (AHN).

### Ciliary contributions to L-LTP and STC

STC is a plasticity mechanism that functions as a coincidence detector, describing the biophysical process whereby transient early LTP transitions into late-LTP (L-LTP), stabilizing memory traces [265–268]. This conversion requires the coincidence of two processes. First, strong convergent excitatory input generates a short-lived synaptic tag [265]. Second, repeated synaptic inputs or significant neuromodulatory events result in cAMP production via a GPCR-adenylate cyclase signaling cascade that activates PKA [266, 269, 270]. Consequently, PKA phosphorylation of CREB transcription factors drives the transcription of IEGs and de novo synthesis of plasticity-related proteins (PRPs), such as Arc and BDNF, that facilitate structural and functional synaptic changes that allow for long-term memory formation [271, 272].

The concentration of GPCR-adenylate cyclase signaling axis in neuronal cilia raises the possibility that ciliary signaling contributes to PKA-dependent STC. Pharmacological antagonism of Gs-coupled 5HT6 in adult and aged rats broadly rescues memory deficits across multiple associative and spatial assays [191, 194, 200, 206]. Conversely, constitutive ablation of Gi-coupled SSTR3 in SSTR3-KO mice led to almost novice performance at one hour after exposure in novel object recognition tasks [211], suggesting an impairment in stabilizing transient memory traces and a potential disruption of STC. Consistent with this, AC3-mediated LTP induced by forskolin stimulation was disrupted in SSTR3-KO mice and during acute SSTR3 antagonism, despite normal electrically evoked LTP [211]. Basal cAMP levels of hippocampal slices were significantly lower in the SSTR3-KO animals [211]. Although it remains unclear whether ciliary cAMP directly regulates STC through effects on IEG transcription, these findings suggest a model in which the ciliary GPCR-adenylate cyclase axis contributes to the neuromodulatory “capture” processes underlying memory stabilization.

AC3 knockout similarly impeded associative memory formation in a time-delayed passive avoidance task, while performance in the classical, non-delayed version of the task remained intact [263, 273]. This pattern indicates that the deficit does not lie in associative learning per se, but in maintaining memory stability across the imposed delay, consistent with impaired L-LTP. This was corroborated by decreased performance of AC3-KO mice in a novel object recognition task 20 min after exposure.

Although ciliary perturbations are empirically associated with memory phenotypes, direct involvement of primary cilia in the STC process remains to be demonstrated. Ciliary cAMP signaling has been shown to generate localized PKA activity, suggesting that perturbations to ciliary signaling could influence PKA-dependent processes. However, there is currently no direct evidence that the ciliary GPCR-AC3-PKA axis regulates STC at synapses, and further investigation is required. For instance, one can examine a potential role of ciliary PKA in driving CREB-dependent transcription. While this has been demonstrated in a non-neuronal context [108], it remains to be determined whether neuronal cilia enriched with neuromodulatory GPCRs can direct local PKA activity toward transcriptional programs that support synaptic plasticity. If established, this would suggest that the primary cilium functions as a signaling amplifier, enhancing the sensitivity and efficiency of neuromodulatory “capture” signals involved in memory stabilization.

### Ciliary roles in brain rhythms and STDP

Engrams can be highly distributed, with a single memory trace spanning across regions including the amygdala, hippocampal-entorhinal cortex, and prefrontal cortex [274, 275]. Such distributed representations require mechanisms to selectively strengthen connectivity across regions, a role fulfilled by STDP. STDP is a form of synaptic plasticity in which near-coincident depolarization of pre- and postsynaptic neurons strengthens synaptic connections [276, 277]. To support this process across spatially distributed networks, the brain relies on rhythmic activity to align neuronal firing with millisecond precision [278, 279]. In particular, phase–amplitude coupling (PAC) between theta and gamma oscillations plays a key role in coordinating interregional communication [279, 280]. The coherence and stability of these oscillations are therefore critical for effective information transfer across brain regions [279–281].

There is emerging evidence for a role of primary cilia in regulating brain rhythms. Strobel et al. (2025) showed that extensive ciliary ablation in adult IFT88 knockout mice significantly attenuated theta-gamma PAC, driven in part by slowed theta oscillations [264]. Similarly, EEG recordings from AC3 knockout mice revealed disrupted brainwave patterns during sleep, which were associated with impaired spatial memory retention in the water maze across training sessions [282]. These findings raise the possibility that cilia-associated signaling mechanisms may contribute to oscillatory coherence and the coordination of distributed neural activity required for memory processing. Such disruptions may compromise the stabilization of distributed engrams, contributing to the associative learning deficits and impaired context retention observed in these models [264, 282].

In addition to facilitating long-term synaptic changes, theta-gamma is also implicated in maintaining persistent ensemble firing to hold information "online" during WM execution, with increased coherence observed in prefrontal cortical neurons during high-demand WM tasks [283]. Although modeling WM deficits in rodents presents challenges due to their short-term (seconds-scale) memory retention, the observed impairments in human ciliopathies, including minute-scale WM deficits in BBS [23, 284], suggest that disrupted oscillatory coordination may have broader consequences for cognitive function. Future studies examining PAC and WM performance in ciliary mutant models will be important for clarifying these relationships.

To understand how brain rhythms enable coordination across distributed regions, it is important to consider how neurons achieve rhythmic alignment at the cellular level. One potential mechanism involves cyclic nucleotide signaling pathways that regulate intrinsic excitability. Increases in intracellular cAMP can modulate ion channel activity, including hyperpolarization-activated cyclic nucleotide-gated (HCN) channels, thereby enhancing the hyperpolarization-activated inward current (I_h_) [285]. I_h_ contributes to the ability of neurons to rhythmically return to an excitable state, selectively amplifying inputs at specific frequency bands, such as theta oscillations [281, 285]. Through these mechanisms, cyclic nucleotide signaling can influence neuronal synchrony and promote coherence across distributed networks.

While it is unknown whether HCN channels may reside in neuronal cilia, the empirical evidence of ciliary involvement in brain rhythm coherence suggests a mechanism that needs to be uncovered. For example, the closely related cyclic nucleotide-gated (CNG) channels are enriched in olfactory cilia, where AC3 knockout reduces cAMP-mediated channel activation, leading to anosmia [273]. This raises the possibility that ciliary cAMP signaling could similarly influence ion channel activity relevant to neuronal rhythmicity. If HCN channels are sufficiently proximal to the primary cilium, ciliary cAMP may contribute to their modulation; however, this remains to be experimentally validated. Notably, ciliary cAMP signaling has been shown to influence overall hippocampal cAMP levels [211], suggesting a potential route through which cilia could affect broader network dynamics.

### Role of cilia-mediated neurogenesis in pattern separation

While STC and STDP describe mechanisms underlying individual engram formation and stabilization, a key requirement for engram specificity and proper function is sparsity [286, 287]. In a high-dimensional space of sensory input, sparsity is crucial for preventing inadvertent simultaneous activation of multiple engrams [286, 287]. This process of distinguishing similar contexts and ensuring activation of the appropriate engram is known as pattern separation. Pattern separation can be illustrated by the rapid discrimination of similar exteroceptive cues. For example, in human visual memory tasks, subjects must distinguish previously encountered objects from highly similar “lures” [288], requiring neural circuits to transform overlapping sensory representations into distinct episodic memory traces.

The dentate gyrus, which receives entorhinal input via the perforant path, is a key site for pattern separation [289]. It supports AHN and utilizes hyper-excitable adult-born granule cells (abGCs) to facilitate the computational task of parsing, distinguishing and allocation of similar inputs [290–292]. During discrimination, the tone that is presented first or is more salient is captured by these abGCs, which in turn engage inhibitory interneuron circuits to produce widespread lateral suppression [293, 294]. This process ensures that subsequent similar input is shunted toward a disparate set of non-inhibited cells, forming a discrete engram with high separability [292]. Disruption of this mechanism has been observed in models with impaired ciliary function. Conditional ablation of IFT20 in adult GFAP + NSCs reduces AHN, leading to deficits in spatial pattern recognition and Barnes maze performance [178], consistent with impaired pattern separation. Similarly, germline BBS1^M390R/M390R^ knock-in, forebrain-specific BBS1 knockout and postnatal-induced BBS8 knockout mouse models commonly exhibit the phenotype of deficits in long-term fear context encoding [295]. In the BBS1^M390R/M390R^ group, initial L-LTP was intact, but hippocampal proliferation was decreased, in both neonatal and adult mice. Subsequently, the behavioral phenotype was rescued using lithium treatment [295], suggesting that impairments in neurogenesis-dependent processes, rather than synaptic plasticity per se, underlie the observed memory deficit. Together, these findings suggest that intact ciliary function, particularly in supporting neurogenesis, may contribute to pattern separation by regulating the generation and integration of adult-born neurons. Disruption of ciliary trafficking machinery may therefore compromise this process, leading to reduced flexibility in encoding similar inputs.

Ultimately, the disparate behavioral deficits, spanning from short-term WM failures in ciliopathic individuals to multi-day associative learning rigidity in knockout animals, cannot be reconciled by viewing the primary cilium merely as a passive sensory appendage. Instead, these phenotypes may be better understood in terms of disruptions to the stability and competition of engrams, the physical substrates of memory. Ciliary signaling may influence multiple processes underlying engram formation and maintenance. These include the stabilization of synaptic tags through sensitivity to transient neuromodulatory signals (STC), the tuning of neuronal resonance to align with interregional brain rhythms (STDP and PAC), and the regulation of adult neurogenesis to support context separation [264, 282]. Through these mechanisms, ciliary function may modulate gain control at multiple levels, influencing thresholds for L-LTP and neurogenesis. In this framework, primary cilia act as regulators of the stability and competition that govern memory formation, reconsolidation, and extinction, linking subcellular signaling processes to higher-order cognitive function.

## Conclusion and outlook

Our review examines the hypothesis that primary cilia function as specialized computational microdomains that integrate intrinsic and extrinsic signals to modulate neuronal firing, plasticity, and circuit dynamics. Testing these roles in mature, behaving systems remains a significant experimental challenge. A primary obstacle is disentangling developmental phenotypes from acute computational deficits, as germline perturbations of ciliary genes often produce widespread structural and circuit-level alterations during early brain development [30, 296]. To address how ciliary signaling influences adult neural function more directly, future studies will benefit from approaches that enable targeted perturbations in post-mitotic neurons.

Developing these acute techniques requires researchers to achieve neuronal subtype specificity, subcellular resolution, and reliable control over signaling pathways. Conditional transgenic lines, such as inducible Cre-loxP models, allow for inducible gene manipulation in postnatal or adult animals, thereby reducing developmental confounds [297]. Viral CRISPR-Cas9 delivery paired with Cre-driver lines provides excellent cell-type and circuit specificity, as well as rapid adaptability for screening targets [298]. However, in vivo editing efficiency is highly variable and may generate a mosaic distribution of functional bi-allelic knockouts versus mono-allelic indels that muddies behavioral readouts [299, 300]. Moreover, the efficient removal of ciliary structure is impeded by the structural persistence and long-lived nature of primary cilia in post-mitotic neurons. Such perturbation is often slow to manifest and may produce indirect or compensatory effects that complicate the establishment of direct causal relationships between cilia disruption and neuronal properties. It is therefore more strategic to target specific ciliary signaling or trafficking modules, which may allow more precise dissection of cilia-dependent functions. Inevitably, gene-targeted approaches also share a fundamental limitation: they eliminate the target protein cell-wide, failing to isolate the cilia specific functions from the somatodendritic signaling.

Achieving subcellular specificity therefore requires targeting genetically encoded sensors and actuators to the primary cilium with high precision and minimal off-target localization. Such targeting strategies include fusion to full-length ciliary GPCRs (e.g., 5HT6) [126], truncated ciliary proteins (e.g., NPHP3) [301], or shorter ciliary targeting sequences (CTS) determined from ciliary proteins. More recently, nanobody-based approaches have been used to direct molecular tools to ciliary proteins with improved specificity [301]. When carefully validated, these approaches enable selective interrogation of ciliary signaling dynamics under defined stimuli [108, 126, 302], including the study of compartmentalized cAMP signaling in both neuronal [164, 165] and non-neuronal contexts [108, 246]. The small geometric volume of the cilium further facilitates relative isolation of signaling events compared to the soma [106, 108], although careful controls are required to ensure minimal spillover.

These genetically encoded cilia-targeted tools can be integrated with circuit-level approaches to investigate how ciliary signaling influences neural activity and behavior. Combining ciliary perturbation with in vivo calcium imaging or electrophysiological recordings in behaving animals offers a way to link subcellular signaling to neuronal dynamics. For example, simultaneous recordings across connected brain regions can reveal how ciliary perturbations influence inter-regional coordination and rhythmic coherence [303, 304]. Analytical frameworks from systems neuroscience, including low-dimensional manifold analysis [305, 306] and attractor dynamics [307–309], may provide quantitative insights into how such perturbations alter ensemble activity and information processing. Ultimately, understanding how ciliary signaling contributes to neural function will require linking molecular perturbations to changes in neuronal activity patterns. Examining how ciliary manipulation alters the encoding and propagation of information across neural circuits represents a key step toward bridging subcellular signaling with systems-level computation.

In parallel, emerging genomic and transcriptomic studies have revealed overlaps between gene networks associated with ciliopathies and those implicated in neurodevelopmental [310] and neuropsychiatric disorders [311]. These findings suggest that ciliary dysfunction may influence gene networks involved in learning and memory. High-throughput approaches such as Perturb-seq [312, 313] provide a powerful platform to systematically interrogate these pathways in vivo, enabling the mapping of transcriptional responses to targeted perturbations. Integrating such approaches with in vivo imaging and spatial transcriptomics may further clarify how altered gene expression in ciliopathies relates to changes in neural circuit function and behavior.

In conclusion, the framework presented in this review positions the primary cilium as a dynamic computational microdomain that integrates and transforms signals across multiple scales of neural organization. By regulating neuronal excitability, rhythmic coordination, and structural plasticity, ciliary signaling may shape how information is encoded, propagated, and stored in the brain. Future efforts combining cilia-targeted molecular tools with systems-level and transcriptomic approaches will be essential for establishing causal links between ciliary signaling and neural computation. Such work will not only clarify the role of primary cilia in normal brain function but may also provide new insights into the mechanisms underlying cognitive dysfunction in ciliopathies and related neurological disorders.

## Data Availability

No datasets were generated or analysed during the current study.

## Abbreviations

abGC: Adult-born granule cell
AC: Adenylate cyclase
AC3: Adenylate cyclase III
AC5: Adenylate cyclase V
AIS: Axon initial segment
AKAP: A-kinase anchoring protein
AgRP: Agouti-related peptide
ARC: Arcuate nucleus
ASD: Autism spectrum disorder
AURKA: Aurora A kinase
BBS: Bardet-Biedl syndrome
cAMP: Cyclic adenosine monophosphate
CREB: CAMP response element-binding protein
CTS: Ciliary targeting sequence
DCN: Deep cerebellar neuron
DREADD: Designer receptors exclusively activated by designer drugs
E/I: Excitation-inhibition
EPSP: Excitatory postsynaptic potential
LTP: Long term potentiation
GNP: Granule neuron progenitor
GPCR: G-protein coupled receptor
GRK2: GPCR kinase 2
hESC: Human embryonic stem cell
HCN: Hyperpolarization-activated cyclic nucleotide-gated
HH: Hedgehog
IEG: Immediate early gene
IFT: Intraflagellar transport
IPSP: Inhibitory postsynaptic potential
JS: Joubert syndrome
L-LTP: Late-long term potentiation
LTP: Long term potentiation
NSC: Neural stem cells
OXTR: Oxytocin receptor
PAC: Phase-amplitude coupling
PKA: Protein kinase A
PRP: Plasticity-related protein
PVH: Paraventricular hypothalamus
RG: Radial glia
SCZ: Schizophrenia
STC: Synaptic tagging and capture
STDP: Spike-time dependent plasticity
WM: Working memory

## References

[1] Guillery RW. Observations of synaptic structures: origins of the neuron doctrine and its current status. Philos Trans R Soc B Biol Sci. 2005;360(1458):1281–307. 10.1098/rstb.2003.1459. (**PubMed PMID: 16147523; PubMed Central PMCID: PMC1569502**). 10.1098/rstb.2003.1459 PMC1569502 16147523

[2] Wang B, Dudko OK. A theory of synaptic transmission. Elife. 2021;10:e73585. 10.7554/eLife.73585. 34970965 10.7554/eLife.73585 PMC8776255

[3] Heiney K, Huse Ramstad O, Fiskum V, Christiansen N, Sandvig A, Nichele S, et al. Criticality, connectivity, and neural disorder: a multifaceted approach to neural computation. Front Comput Neurosci. 2021. 10.3389/fncom.2021.611183. 33643017 10.3389/fncom.2021.611183 PMC7902700

[4] Cajal SRY, Azoulay DL, swanson N, Swanson larry W. Histology Of The Nervous System: Of Man And Vertebrates [Internet]. Oxford University Press; 1995 [cited 2026 Apr 7]. 10.1093/oso/9780195074017.001.0001 10.1093/oso/9780195074017.001.0001

[5] Luo L. Architectures of neuronal circuits. Science. 2021;373(6559):eabg7285. 10.1126/science.abg7285. 34516844 10.1126/science.abg7285 PMC8916593

[6] Kathuria A, Lopez-Lengowski K, Watmuff B, Karmacharya R. Morphological and transcriptomic analyses of stem cell-derived cortical neurons reveal mechanisms underlying synaptic dysfunction in schizophrenia. Genome Med. 2023;15(1):58. 10.1186/s13073-023-01203-5. 37507766 10.1186/s13073-023-01203-5 PMC10375745

[7] Berdenis van Berlekom A, Muflihah CH, Snijders GJLJ, MacGillavry HD, Middeldorp J, Hol EM, et al. Synapse pathology in schizophrenia: a meta-analysis of postsynaptic elements in postmortem brain studies. Schizophr Bull. 2020;46(2):374–86. 10.1093/schbul/sbz060. (**PubMed PMID: 31192350; PubMed Central PMCID: PMC7442385**). 31192350 10.1093/schbul/sbz060 PMC7442385

[8] Alemany-González M, Gener T, Nebot P, Vilademunt M, Dierssen M, Puig MV. Prefrontal-hippocampal functional connectivity encodes recognition memory and is impaired in intellectual disability. Proc Natl Acad Sci U S A. 2020;117(21):11788–98. 10.1073/pnas.1921314117. (**PubMed PMID: 32393630; PubMed Central PMCID: PMC7261130**). 32393630 10.1073/pnas.1921314117 PMC7261130

[9] Asiminas A, Booker SA, Dando OR, Kozic Z, Arkell D, Inkpen FH, et al. Experience-dependent changes in hippocampal spatial activity and hippocampal circuit function are disrupted in a rat model of Fragile X Syndrome. Mol Autism. 2022;13(1):49. 10.1186/s13229-022-00528-z. 36536454 10.1186/s13229-022-00528-z PMC9764562

[10] Smith-Hicks CL, Cai P, Savonenko AV, Reeves RH, Worley PF. Increased sparsity of hippocampal CA1 neuronal ensembles in a mouse model of Down Syndrome assayed by Arc expression. Front Neural Circuits. 2017;11:6. 10.3389/fncir.2017.00006. (**PubMed PMID: 28217086; PubMed Central PMCID: PMC5289947**). 28217086 10.3389/fncir.2017.00006 PMC5289947

[11] Tsang SH, Aycinena ARP, Sharma T. Ciliopathy: Bardet-Biedl Syndrome. Adv Exp Med Biol. 2018;1085:171–4. 10.1007/978-3-319-95046-4_33. (**PubMed PMID: 30578506**). 30578506 10.1007/978-3-319-95046-4_33

[12] Parisi MA. The molecular genetics of Joubert syndrome and related ciliopathies: the challenges of genetic and phenotypic heterogeneity. Transl Sci Rare Dis. 2019;4(1–2):25–49. 10.3233/TRD-190041. (**PubMed PMID: 31763177; PubMed Central PMCID: PMC6864416**). 31763177 10.3233/TRD-190041 PMC6864416

[13] Franco B, Thauvin-Robinet C. Update on oral-facial-digital syndromes (OFDS). Cilia. 2016;5(1):12. 10.1186/s13630-016-0034-4. 27141300 10.1186/s13630-016-0034-4 PMC4852435

[14] Asadollahi R, Strauss JE, Zenker M, Beuing O, Edvardson S, Elpeleg O, et al. Clinical and experimental evidence suggest a link between KIF7 and C5orf42-related ciliopathies through Sonic Hedgehog signaling. Eur J Hum Genet. 2018;26(2):197–209. 10.1038/s41431-017-0019-9. (**PubMed PMID: 29321670; PubMed Central PMCID: PMC5839020.**). 29321670 10.1038/s41431-017-0019-9 PMC5839020

[15] Maria BL, Hoang KBN, Tusa RJ, Mancuso AA, Hamed LM, Quisling RG, et al. ‘Joubert syndrome’ revisited: key ocular motor signs with magnetic resonance imaging correlation. J Child Neurol. 1997;12(7):423–30. 10.1177/088307389701200703. 9373798 10.1177/088307389701200703

[16] Summers AC, Snow J, Wiggs E, Liu AG, Toro C, Poretti A, et al. Neuropsychological phenotypes of 76 individuals with Joubert syndrome evaluated at a single center. Am J Med Genet A. 2017;173(7):1796–812. 10.1002/ajmg.a.38272. 28497568 10.1002/ajmg.a.38272 PMC5682233

[17] Rooryck C, Pelras S, Chateil JF, Cances C, Arveiler B, Verloes A, et al. Bardet-Biedl syndrome and brain abnormalities. Neuropediatrics. 2007;38(1):5–9. 10.1055/s-2007-981466. (**PubMed PMID: 17607597.**). 17607597 10.1055/s-2007-981466

[18] Barnett S, Reilly S, Carr L, Ojo I, Beales PL, Charman T. Behavioural phenotype of Bardet-Biedl syndrome. J Med Genet. 2002;39(12):e76–e76. 10.1136/jmg.39.12.e76. (**PubMed PMID: 12471214.**). 12471214 10.1136/jmg.39.12.e76 PMC1757216

[19] Spahiu L, Behluli E, Grajçevci-Uka V, Liehr T, Temaj G. Joubert syndrome: molecular basis and treatment. J Mother Child. 2022;26(1):118–23. 10.34763/jmotherandchild.20222601.d-22-00034. (**PubMed PMID: 36803942; PubMed Central PMCID: PMC10032320.**). 36803942 10.34763/jmotherandchild.20222601.d-22-00034 PMC10032320

[20] Fennell EB, Gitten JC, Dede DE, Maria BL. Cognition, behavior, and development in Joubert syndrome. J Child Neurol. 1999;14(9):592–6. 10.1177/088307389901400907. 10488904 10.1177/088307389901400907

[21] Poretti A, Gerner GJ. Neurocognitive functions and behavior in Joubert syndrome. Pediatr Neurol Briefs. 2016;30(12):47. 10.15844/pedneurbriefs-30-12-3. (**PubMed PMID: 27956815; PubMed Central PMCID: PMC5133046.**). 27956815 10.15844/pedneurbriefs-30-12-3 PMC5133046

[22] Bulgheroni S, D’Arrigo S, Signorini S, Briguglio M, Di Sabato ML, Casarano M, et al. Cognitive, adaptive, and behavioral features in Joubert syndrome. Am J Med Genet A. 2016;170(12):3115–24. 10.1002/ajmg.a.37938. (**PubMed PMID: 27530364.**). 27530364 10.1002/ajmg.a.37938

[23] Brinckman DD, Keppler-Noreuil KM, Blumhorst C, Biesecker LG, Sapp JC, Johnston JJ, et al. Cognitive, sensory, and psychosocial characteristics in patients with Bardet-Biedl syndrome. Am J Med Genet A. 2013;0(12):2964–71. 10.1002/ajmg.a.36245. (**PubMed PMID: 24194441; PubMed Central PMCID: PMC4419571.**). 10.1002/ajmg.a.36245 PMC4419571 24194441

[24] Beales PL, Elcioglu N, Woolf AS, Parker D, Flinter FA. New criteria for improved diagnosis of Bardet-Biedl syndrome: results of a population survey. J Med Genet. 1999;36(6):437–46. 10.1136/jmg.36.6.437. 10874630 PMC1734378

[25] Kerr EN, Bhan A, Héon E. Exploration of the cognitive, adaptive and behavioral functioning of patients affected with Bardet–Biedl syndrome. Clin Genet. 2016;89(4):426–33. 10.1111/cge.12614. 25988237 10.1111/cge.12614

[26] Gusev A, Mancuso N, Won H, Kousi M, Finucane HK, Reshef Y, et al. Transcriptome-wide association study of schizophrenia and chromatin activity yields mechanistic disease insights. Nat Genet. 2018;50(4):538–48. 10.1038/s41588-018-0092-1. 29632383 10.1038/s41588-018-0092-1 PMC5942893

[27] Monroe TO, Garrett ME, Kousi M, Rodriguiz RM, Moon S, Bai Y, et al. PCM1 is necessary for focal ciliary integrity and is a candidate for severe schizophrenia. Nat Commun. 2020;11(1):5903. 10.1038/s41467-020-19637-5. 33214552 10.1038/s41467-020-19637-5 PMC7677393

[28] Kostyanovskaya E, Lasser MC, Wang B, Schmidt J, Bader E, Buteo C, et al. Convergence of autism proteins at the cilium. BioRxiv Prepr Serv Biol. 2025;2024.12.05.626924. 10.1101/2024.12.05.626924**PubMed PMID: 39677731; PubMed Central PMCID: PMC11643032**.

[29] Alhassen W, Chen S, Vawter M, Robbins BK, Nguyen H, Myint TN, et al. Patterns of cilia gene dysregulations in major psychiatric disorders. Prog Neuropsychopharmacol Biol Psychiatry. 2021;109:110255. 10.1016/j.pnpbp.2021.110255. 33508383 10.1016/j.pnpbp.2021.110255 PMC9121176

[30] Bangs F, Anderson KV. Primary cilia and mammalian Hedgehog signaling. Cold Spring Harb Perspect Biol. 2017;9(5):a028175. 10.1101/cshperspect.a028175. 27881449 10.1101/cshperspect.a028175 PMC5411695

[31] Wang L, Hou S, Han YG. Hedgehog signaling promotes basal progenitor expansion and the growth and folding of the neocortex. Nat Neurosci. 2016;19(7):888–96. 10.1038/nn.4307. 27214567 10.1038/nn.4307 PMC4925239

[32] Oishi K, Nakajima K, Motoyama J. Activation of Sonic Hedgehog signaling promotes differentiation of cortical layer 4 neurons via regulation of their cell positioning. J Dev Biol. 2022;10(4):50. 10.3390/jdb10040050. 36547472 10.3390/jdb10040050 PMC9787542

[33] Somaiya RD, Stebbins K, Gingrich EC, Xie H, Campbell JN, Garcia ADR, et al. Sonic hedgehog-dependent recruitment of GABAergic interneurons into the developing visual thalamus. Elife. 2022;11:e79833. 10.7554/eLife.79833. 36342840 10.7554/eLife.79833 PMC9640189

[34] He W, Cui L, Zhang C, Zhang X, He J, Xie Y. Sonic Hedgehog promotes neurite outgrowth of primary cortical neurons through up-regulating BDNF expression. Neurochem Res. 2016;41(4):687–95. 10.1007/s11064-015-1736-5. 26459360 10.1007/s11064-015-1736-5

[35] Dumoulin A, Wilson NH, Tucker KL, Stoeckli ET. A cell-autonomous role for primary cilium-mediated signaling in long-range commissural axon guidance. Development. 2024;151(17):dev202788. 10.1242/dev.202788. (**PubMed PMID: 39157903; PubMed Central PMCID: PMC11423920.**). 39157903 10.1242/dev.202788 PMC11423920

[36] Sterpka A, Chen X. Neuronal and astrocytic primary cilia in the mature brain. Pharmacol Res. 2018;137:114–21. 10.1016/j.phrs.2018.10.002. 30291873 10.1016/j.phrs.2018.10.002 PMC6410375

[37] Gonzalez-Hernandez AJ, Munguba H, Levitz J. Emerging modes of regulation of neuromodulatory G protein-coupled receptors. Trends Neurosci. 2024;47(8):635–50. 10.1016/j.tins.2024.05.008. 38862331 10.1016/j.tins.2024.05.008 PMC11324403

[38] Lohse MJ, Bock A, Zaccolo M. G Protein-Coupled Receptor signaling: new insights define cellular nanodomains. Annu Rev Pharmacol Toxicol. 2024;64:387–415. 10.1146/annurev-pharmtox-040623-115054. (**PubMed PMID: 37683278.**). 37683278 10.1146/annurev-pharmtox-040623-115054

[39] Sun Y, Hasbi A, George SR. G Protein-Coupled Receptor heteromers in brain: functional and therapeutic importance in neuropsychiatric disorders. Annu Rev Pharmacol Toxicol. 2025;65(1):215–36. 10.1146/annurev-pharmtox-061724-080727. (**PubMed PMID: 39847466.**). 39847466 10.1146/annurev-pharmtox-061724-080727

[40] Benenson Y. Biomolecular computing systems: principles, progress and potential. Nat Rev Genet. 2012;13(7):455–68. 10.1038/nrg3197. 22688678 10.1038/nrg3197

[41] Bray D. Protein molecules as computational elements in living cells. Nature. 1995;376(6538):307–12. 10.1038/376307a0. 7630396 10.1038/376307a0

[42] Dayan P, Abbott LF, Abbott LF. Theoretical neuroscience: computational and mathematical modeling of neural systems. First paperback ed. Cambridge, Mass.: MIT Press; 2005. 460 p. (Computational neuroscience).

[43] Carandini M. From circuits to behavior: a bridge too far? Nat Neurosci. 2012;15(4):507–9. 10.1038/nn.3043. 22449960 10.1038/nn.3043

[44] Silver RA. Neuronal arithmetic. Nat Rev Neurosci. 2010;11(7):474–89. 10.1038/nrn2864. 20531421 10.1038/nrn2864 PMC4750293

[45] Alcami P, El Hady A. Axonal computations. Front Cell Neurosci. 2019;13:413. 10.3389/fncel.2019.00413. (**PubMed PMID: 31619963; PubMed Central PMCID: PMC6759653.**). 31619963 10.3389/fncel.2019.00413 PMC6759653

[46] McDonnell MD, Ikeda S, Manton JH. An introductory review of information theory in the context of computational neuroscience. Biol Cybern. 2011;105(1):55–70. 10.1007/s00422-011-0451-9. 21792610 10.1007/s00422-011-0451-9

[47] Chung S, Abbott LF. Neural population geometry: an approach for understanding biological and artificial neural networks. Curr Opin Neurobiol. 2021;70:137–44. 10.1016/j.conb.2021.10.010. 34801787 10.1016/j.conb.2021.10.010 PMC10695674

[48] Dimitrov AG, Lazar AA, Victor JD. Information theory in neuroscience. J Comput Neurosci. 2011;30(1):1–5. 10.1007/s10827-011-0314-3. (**PubMed PMID: 21279429; PubMed Central PMCID: PMC3736735.**). 21279429 10.1007/s10827-011-0314-3 PMC3736735

[49] Yu A, Lau AY. Glutamate and glycine binding to the NMDA receptor. Structure Lond Engl. 2018;26(7):1035-1043.e2. 10.1016/j.str.2018.05.004. (**PubMed PMID: 29887499; PubMed Central PMCID: PMC6031449.**). 10.1016/j.str.2018.05.004 PMC6031449 29887499

[50] Abbott LF, Regehr WG. Synaptic computation. Nature. 2004;431(7010):796–803. 10.1038/nature03010. 15483601 10.1038/nature03010

[51] Fischer L, Mojica Soto-Albors R, Tang VD, Bicknell B, Grienberger C, Francioni V, et al. Dendritic mechanisms for in vivo neural computations and behavior. J Neurosci. 2022;42(45):8460–7. 10.1523/JNEUROSCI.1132-22.2022. (**PubMed PMID: 36351832; PubMed Central PMCID: PMC9665914**). 36351832 10.1523/JNEUROSCI.1132-22.2022 PMC9665914

[52] London M, Häusser M. Dendritic computation. Annu Rev Neurosci. 2005;28(1):503–32. 10.1146/annurev.neuro.28.061604.135703. 16033324 10.1146/annurev.neuro.28.061604.135703

[53] Branco T, Häusser M. Synaptic integration gradients in single cortical pyramidal cell dendrites. Neuron. 2011;69(5):885–92. 10.1016/j.neuron.2011.02.006. (**PubMed PMID: 21382549; PubMed Central PMCID: PMC6420135**). 21382549 10.1016/j.neuron.2011.02.006 PMC6420135

[54] Evans MD, Dumitrescu AS, Kruijssen DLH, Taylor SE, Grubb MS. Rapid modulation of axon initial segment length influences repetitive spike firing. Cell Rep. 2015;13(6):1233–45. 10.1016/j.celrep.2015.09.066. (**PubMed PMID: 26526995**). 26526995 10.1016/j.celrep.2015.09.066 PMC4646840

[55] Kole MHP, Stuart GJ. Signal processing in the axon initial segment. Neuron. 2012;73(2):235–47. 10.1016/j.neuron.2012.01.007. (**PubMed PMID: 22284179**). 22284179 10.1016/j.neuron.2012.01.007

[56] Foust A, Popovic M, Zecevic D, McCormick DA. Action potentials initiate in the axon initial segment and propagate through axon collaterals reliably in cerebellar Purkinje neurons. J Neurosci. 2010;30(20):6891–902. 10.1523/JNEUROSCI.0552-10.2010. (**PubMed PMID: 20484631; PubMed Central PMCID: PMC2990270**). 20484631 10.1523/JNEUROSCI.0552-10.2010 PMC2990270

[57] Kole MHP, Letzkus JJ, Stuart GJ. Axon initial segment Kv1 channels control axonal action potential waveform and synaptic efficacy. Neuron. 2007;55(4):633–47. 10.1016/j.neuron.2007.07.031. (**PubMed PMID: 17698015**). 17698015 10.1016/j.neuron.2007.07.031

[58] Meeks JP, Mennerick S. Action potential initiation and propagation in CA3 pyramidal axons. J Neurophysiol. 2007;97(5):3460–72. 10.1152/jn.01288.2006. (**PubMed PMID: 17314237**). 17314237 10.1152/jn.01288.2006

[59] Palmer LM, Stuart GJ. Site of action potential initiation in layer 5 pyramidal neurons. J Neurosci. 2006;26(6):1854–63. 10.1523/JNEUROSCI.4812-05.2006. (**PubMed PMID: 16467534; PubMed Central PMCID: PMC6793621**). 16467534 10.1523/JNEUROSCI.4812-05.2006 PMC6793621

[60] Smith PM, Ferguson AV. Neurophysiology of hunger and satiety. Dev Disabil Res Rev. 2008;14(2):96–104. 10.1002/ddrr.13. (**PubMed PMID: 18646014**). 18646014 10.1002/ddrr.13

[61] Andermann ML, Lowell BB. Toward a wiring diagram understanding of appetite control. Neuron. 2017;95(4):757–78. 10.1016/j.neuron.2017.06.014. 28817798 10.1016/j.neuron.2017.06.014 PMC5657399

[62] Jais A, Brüning JC. Arcuate nucleus-dependent regulation of metabolism—Pathways to obesity and diabetes mellitus. Endocr Rev. 2022;43(2):314–28. 10.1210/endrev/bnab025. 34490882 10.1210/endrev/bnab025 PMC8905335

[63] Paisdzior S, Dimitriou IM, Schöpe PC, Annibale P, Scheerer P, Krude H, et al. Differential signaling profiles of MC4R mutations with three different ligands. Int J Mol Sci. 2020;21(4):1224. 10.3390/ijms21041224. (**PubMed PMID: 32059383; PubMed Central PMCID: PMC7072973**). 32059383 10.3390/ijms21041224 PMC7072973

[64] D’Agostino G, Diano S. Alpha-melanocyte stimulating hormone: production and degradation. J Mol Med Berl. 2010;88(12):1195–201. 10.1007/s00109-010-0651-0. (**PubMed PMID: 20617297; PubMed Central PMCID: PMC3936413**). 20617297 10.1007/s00109-010-0651-0 PMC3936413

[65] Nijenhuis WA, Oosterom J, Adan RA. AgRP(83-132) acts as an inverse agonist on the human-melanocortin-4 receptor. Mol Endocrinol Baltim. 2001;15(1):164–71. 10.1210/mend.15.1.0578. (**PubMed PMID: 11145747**). 10.1210/mend.15.1.0578 11145747

[66] Ollmann MM, Wilson BD, Yang YK, Kerns JA, Chen Y, Gantz I, et al. Antagonism of central melanocortin receptors in vitro and in vivo by agouti-related protein. Science. 1997;278(5335):135–8. 10.1126/science.278.5335.135. (**PubMed PMID: 9311920**). 9311920 10.1126/science.278.5335.135

[67] Gold JI, Shadlen MN. The neural basis of decision making. Annu Rev Neurosci. 2007;30(1):535–74. 10.1146/annurev.neuro.29.051605.113038. 17600525 10.1146/annurev.neuro.29.051605.113038

[68] Miller EK, Cohen JD. An integrative theory of prefrontal cortex function. Annu Rev Neurosci. 2001;24(1):167–202. 10.1146/annurev.neuro.24.1.167. 11283309 10.1146/annurev.neuro.24.1.167

[69] Hanks TD, Summerfield C. Perceptual decision making in rodents, monkeys, and humans. Neuron. 2017;93(1):15–31. 10.1016/j.neuron.2016.12.003. (**PubMed PMID: 28056343**). 28056343 10.1016/j.neuron.2016.12.003

[70] Balleine BW, O’Doherty JP. Human and rodent homologies in action control: corticostriatal determinants of goal-directed and habitual action. Neuropsychopharmacology. 2010;35(1):48–69. 10.1038/npp.2009.131. (**PubMed PMID: 19776734; PubMed Central PMCID: PMC3055420.**). 19776734 10.1038/npp.2009.131 PMC3055420

[71] Dani VS, Chang Q, Maffei A, Turrigiano GG, Jaenisch R, Nelson SB. Reduced cortical activity due to a shift in the balance between excitation and inhibition in a mouse model of Rett Syndrome. Proc Natl Acad Sci U S A. 2005;102(35):12560–5. 10.1073/pnas.0506071102. 16116096 10.1073/pnas.0506071102 PMC1194957

[72] Lam NH, Borduqui T, Hallak J, Roque A, Anticevic A, Krystal JH, et al. Effects of altered excitation-inhibition balance on decision making in a cortical circuit model. J Neurosci. 2022;42(6):1035–53. 10.1523/JNEUROSCI.1371-20.2021. (**PubMed PMID: 34887320**). 34887320 10.1523/JNEUROSCI.1371-20.2021 PMC8824494

[73] Marín-Burgin A, Mongiat LA, Pardi MB, Schinder AF. Unique processing during a period of high excitation/inhibition balance in adult-born neurons. Science. 2012;335(6073):1238–42. 10.1126/science.1214956. 22282476 10.1126/science.1214956 PMC3385415

[74] Yizhar O, Fenno LE, Prigge M, Schneider F, Davidson TJ, O’Shea DJ, et al. Neocortical excitation/inhibition balance in information processing and social dysfunction. Nature. 2011;477(7363):171–8. 10.1038/nature10360. 21796121 10.1038/nature10360 PMC4155501

[75] Stief F, Zuschratter W, Hartmann K, Schmitz D, Draguhn A. Enhanced synaptic excitation–inhibition ratio in hippocampal interneurons of rats with temporal lobe epilepsy. Eur J Neurosci. 2007;25(2):519–28. 10.1111/j.1460-9568.2006.05296.x. 17284194 10.1111/j.1460-9568.2006.05296.x

[76] Sohal VS, Rubenstein JLR. Excitation-inhibition balance as a framework for investigating mechanisms in neuropsychiatric disorders. Mol Psychiatry. 2019;24(9):1248–57. 10.1038/s41380-019-0426-0. 31089192 10.1038/s41380-019-0426-0 PMC6742424

[77] Nachury MV. How do cilia organize signalling cascades? Philos Trans R Soc Lond B Biol Sci. 2014;369(1650):20130465. 10.1098/rstb.2013.0465. 25047619 10.1098/rstb.2013.0465 PMC4113109

[78] Lacey SE, Pigino G. The intraflagellar transport cycle. Nat Rev Mol Cell Biol. 2025;26(3):175–92. 10.1038/s41580-024-00797-x. 39537792 10.1038/s41580-024-00797-x

[79] Tian X, Zhao H, Zhou J. Organization, functions, and mechanisms of the BBSome in development, ciliopathies, and beyond. Elife. 2023;12:e87623. 10.7554/eLife.87623. 37466224 10.7554/eLife.87623 PMC10356136

[80] Park K, Leroux MR. Composition, organization and mechanisms of the transition zone, a gate for the cilium. EMBO Rep. 2022;23(12):e55420. 10.15252/embr.202255420. 36408840 10.15252/embr.202255420 PMC9724682

[81] Macarelli V, Leventea E, Merkle FT. Regulation of the length of neuronal primary cilia and its potential effects on signalling. Trends Cell Biol. 2023;33(11):979–90. 10.1016/j.tcb.2023.05.005. (**PubMed PMID: 37302961; PubMed Central PMCID: PMC7615206**). 37302961 10.1016/j.tcb.2023.05.005 PMC7615206

[82] Schou KB, Pedersen LB, Christensen ST. Ins and outs of GPCR signaling in primary cilia. EMBO Rep. 2015;16(9):1099–113. 10.15252/embr.201540530. (**PubMed PMID: 26297609; PubMed Central PMCID: PMC4576980**). 26297609 10.15252/embr.201540530 PMC4576980

[83] Mykytyn K, Askwith C. G-Protein-Coupled Receptor signaling in cilia. Cold Spring Harb Perspect Biol. 2017;9(9):a028183. 10.1101/cshperspect.a028183. (**PubMed PMID: 28159877; PubMed Central PMCID: PMC5585845**). 28159877 10.1101/cshperspect.a028183 PMC5585845

[84] Christensen ST, Clement CA, Satir P, Pedersen LB. Primary cilia and coordination of receptor tyrosine kinase (RTK) signalling. J Pathol. 2012;226(2):172–84. 10.1002/path.3004. 21956154 10.1002/path.3004 PMC4294548

[85] Yeh C, Li A, Chuang JZ, Saito M, Cáceres A, Sung CH. IGF-1 activates a cilium-localized non-canonical Gβγ signaling pathway that regulates cell cycle progression. Dev Cell. 2013;26(4):358–68. 10.1016/j.devcel.2013.07.014. (**PubMed PMID: 23954591; PubMed Central PMCID: PMC3790638**). 23954591 10.1016/j.devcel.2013.07.014 PMC3790638

[86] Clement DL, Mally S, Stock C, Lethan M, Satir P, Schwab A, et al. PDGFRα signaling in the primary cilium regulates NHE1-dependent fibroblast migration via coordinated differential activity of MEK1/2-ERK1/2-p90RSK and AKT signaling pathways. J Cell Sci. 2012. 10.1242/jcs.116426. 23264740 10.1242/jcs.116426 PMC4481637

[87] Schneider L, Clement CA, Teilmann SC, Pazour GJ, Hoffmann EK, Satir P, et al. PDGFRαα signaling is regulated through the primary cilium in fibroblasts. Curr Biol. 2005;15(20):1861–6. 10.1016/j.cub.2005.09.012. 16243034 10.1016/j.cub.2005.09.012

[88] Phua SC, Lin YC, Inoue T. An intelligent nano-antenna: primary cilium harnesses TRP channels to decode polymodal stimuli. Cell Calcium. 2015;58(4):415–22. 10.1016/j.ceca.2015.03.005. 25828566 10.1016/j.ceca.2015.03.005 PMC4564334

[89] Qiu L, LeBel RP, Storm DR, Chen X. Type 3 adenylyl cyclase: a key enzyme mediating the cAMP signaling in neuronal cilia. Int J Physiol Pathophysiol Pharmacol. 2016;8(3):95–108 (**PubMed PMID: 27785336; PubMed Central PMCID: PMC5078481**). 27785336 PMC5078481

[90] Jacoby M, Cox JJ, Gayral S, Hampshire DJ, Ayub M, Blockmans M, et al. INPP5E mutations cause primary cilium signaling defects, ciliary instability and ciliopathies in human and mouse. Nat Genet. 2009;41(9):1027–31. 10.1038/ng.427. 19668215 10.1038/ng.427

[91] Phua SC, Chiba S, Suzuki M, Su E, Roberson EC, Pusapati GV, et al. Dynamic remodeling of membrane composition drives cell cycle through primary cilia excision. Cell. 2017;168(1):264-279.e15. 10.1016/j.cell.2016.12.032. (**PubMed PMID: 28086093**). 28086093 10.1016/j.cell.2016.12.032 PMC5660509

[92] Mick DU, Rodrigues RB, Leib RD, Adams CM, Chien AS, Gygi SP, et al. Proteomics of primary cilia by proximity labeling. Dev Cell. 2015;35(4):497–512. 10.1016/j.devcel.2015.10.015. (**PubMed PMID: 26585297; PubMed Central PMCID: PMC4662609**). 26585297 10.1016/j.devcel.2015.10.015 PMC4662609

[93] Haycraft CJ, Banizs B, Aydin-Son Y, Zhang Q, Michaud EJ, Yoder BK. Gli2 and Gli3 localize to cilia and require the intraflagellar transport protein Polaris for processing and function. PLoS Genet. 2005;1(4):e53. 10.1371/journal.pgen.0010053. 16254602 10.1371/journal.pgen.0010053 PMC1270009

[94] Purvis JE, Lahav G. Encoding and decoding cellular information through signaling dynamics. Cell. 2013;152(5):945–56. 10.1016/j.cell.2013.02.005. 23452846 10.1016/j.cell.2013.02.005 PMC3707615

[95] Kholodenko BN, Hancock JF, Kolch W. Signalling ballet in space and time. Nat Rev Mol Cell Biol. 2010;11(6):414–26. 10.1038/nrm2901. 20495582 10.1038/nrm2901 PMC2977972

[96] Anvarian Z, Mykytyn K, Mukhopadhyay S, Pedersen LB, Christensen ST. Cellular signalling by primary cilia in development, organ function and disease. Nat Rev Nephrol. 2019;15(4):199–219. 10.1038/s41581-019-0116-9. 30733609 10.1038/s41581-019-0116-9 PMC6426138

[97] Gigante ED, Caspary T. Signaling in the primary cilium through the lens of the Hedgehog pathway. WIREs Dev Biol. 2020;9(6):e377. 10.1002/wdev.377. 10.1002/wdev.377 PMC7444278 32084300

[98] Ho EK, Stearns T. Hedgehog signaling and the primary cilium: implications for spatial and temporal constraints on signaling. Development. 2021;148(9):dev195552. 10.1242/dev.195552. 33914866 10.1242/dev.195552 PMC8126410

[99] Mukhopadhyay S, Wen X, Ratti N, Loktev A, Rangell L, Scales SJ, et al. The ciliary G-protein-coupled receptor Gpr161 negatively regulates the Sonic Hedgehog pathway via cAMP signaling. Cell. 2013;152(1–2):210–23. 10.1016/j.cell.2012.12.026. 23332756 10.1016/j.cell.2012.12.026

[100] Garcia-Gonzalo FR, Phua SC, Roberson EC, Garcia G, Abedin M, Schurmans S, et al. Phosphoinositides regulate ciliary protein trafficking to modulate Hedgehog signaling. Dev Cell. 2015;34(4):400–9. 10.1016/j.devcel.2015.08.001. 26305592 10.1016/j.devcel.2015.08.001 PMC4557815

[101] Wang B, Fallon JF, Beachy PA. Hedgehog-regulated processing of Gli3 produces an anterior/posterior repressor gradient in the developing vertebrate limb. Cell. 2000;100(4):423–34. 10.1016/S0092-8674(00)80678-9. (**PubMed PMID: 10693759.**). 10693759 10.1016/s0092-8674(00)80678-9

[102] Zhou M, Jiang J. Gli phosphorylation code in Hedgehog signal transduction. Front Cell Dev Biol. 2022;10:846927. 10.3389/fcell.2022.846927. 35186941 10.3389/fcell.2022.846927 PMC8855225

[103] Bachmann VA, Mayrhofer JE, Ilouz R, Tschaikner P, Raffeiner P, Röck R, et al. Gpr161 anchoring of PKA consolidates GPCR and cAMP signaling. Proc Natl Acad Sci. 2016;113(28):7786–91. 10.1073/pnas.1608061113. 27357676 10.1073/pnas.1608061113 PMC4948347

[104] Paillard T, Allam A, Doulazmi M, Hautefeuille M, Fouquet C, Sarde L, et al. GPR161 mechanosensitivity at the primary cilium drives neuronal saltatory migration. Sci Adv. 2025;11(31):eadx3846. 10.1126/sciadv.adx3846. 40737401 10.1126/sciadv.adx3846 PMC12309678

[105] Walker MF, Zhang J, Steiner W, Ku PI, Zhu JF, Michaelson Z, et al. GRK2 kinases in the primary cilium initiate SMOOTHENED-PKA signaling in the Hedgehog cascade. PLoS Biol. 2024;22(8):e3002685. 10.1371/journal.pbio.3002685. 39138140 10.1371/journal.pbio.3002685 PMC11322411

[106] Moore BS, Stepanchick AN, Tewson PH, Hartle CM, Zhang J, Quinn AM, et al. Cilia have high cAMP levels that are inhibited by Sonic Hedgehog-regulated calcium dynamics. Proc Natl Acad Sci U S A. 2016;113(46):13069–74. 10.1073/pnas.1602393113. (**PubMed PMID: 27799542; PubMed Central PMCID: PMC5135322.**). 27799542 10.1073/pnas.1602393113 PMC5135322

[107] Shim S, Goyal R, Panoutsopoulos AA, Balashova OA, Lee D, Borodinsky LN. Calcium dynamics at the neural cell primary cilium regulate Hedgehog signaling–dependent neurogenesis in the embryonic neural tube. Proc Natl Acad Sci U S A. 2023;120(23):e2220037120. 10.1073/pnas.2220037120. 37252980 10.1073/pnas.2220037120 PMC10266006

[108] Truong ME, Bilekova S, Choksi SP, Li W, Bugaj LJ, Xu K, et al. Vertebrate cells differentially interpret ciliary and extraciliary cAMP. Cell. 2021;184(11):2911-2926.e18. 10.1016/j.cell.2021.04.002. (**PubMed PMID: 33932338; PubMed Central PMCID: PMC8450001.**). 33932338 10.1016/j.cell.2021.04.002 PMC8450001

[109] Pazour GJ, Dickert BL, Vucica Y, Seeley ES, Rosenbaum JL, Witman GB, et al. *Chlamydomonas IFT* 88 and its Mouse homologue, Polycystic Kidney Disease gene *Tg* 737, are required for assembly of cilia and flagella. J Cell Biol. 2000;151(3):709–18. 10.1083/jcb.151.3.709. 11062270 10.1083/jcb.151.3.709 PMC2185580

[110] Keady BT, Samtani R, Tobita K, Tsuchya M, San Agustin JT, Follit JA, et al. IFT25 links the signal-dependent movement of Hedgehog components to intraflagellar transport. Dev Cell. 2012;22(5):940–51. 10.1016/j.devcel.2012.04.009. 22595669 10.1016/j.devcel.2012.04.009 PMC3366633

[111] Eguether T, San Agustin JT, Keady BT, Jonassen JA, Liang Y, Francis R, et al. IFT27 links the BBSome to IFT for maintenance of the ciliary signaling compartment. Dev Cell. 2014;31(3):279–90. 10.1016/j.devcel.2014.09.011. (**PubMed PMID: 25446516; PubMed Central PMCID: PMC4254547.**). 25446516 10.1016/j.devcel.2014.09.011 PMC4254547

[112] Nozaki S, Castro Araya RF, Katoh Y, Nakayama K. Requirement of IFT-B–BBSome complex interaction in export of GPR161 from cilia. Biol Open. 2019. 10.1242/bio.043786. 31471295 10.1242/bio.043786 PMC6777367

[113] Nishat K, Klug Z, Faimma Mia J, Stump SM, Cherry Liu Y. IFT139 regulates Hedgehog signaling and cilia structure through ciliary protein localization. Biol Open. 2025;14(10):bio062040. 10.1242/bio.062040. 41099179 10.1242/bio.062040 PMC12570150

[114] Stuck MW, Gupta M, Knutson LN, Desai PB, Robert KL, Anuszczyk JJ, et al. Ift43 Controls the Ciliary Levels of Gli2 and Gli3 [Internet]. Cell Biology; 2026 [cited 2026 Apr 25]. http://biorxiv.org/lookup/doi/10.64898/2026.01.14.699321 10.64898/2026.01.14.699321

[115] Loktev AV, Zhang Q, Beck JS, Searby CC, Scheetz TE, Bazan JF, et al. A BBSome subunit links ciliogenesis, microtubule stability, and acetylation. Dev Cell. 2008;15(6):854–65. 10.1016/j.devcel.2008.11.001. (**PubMed PMID: 19081074.**). 19081074 10.1016/j.devcel.2008.11.001

[116] Berbari NF, Lewis JS, Bishop GA, Askwith CC, Mykytyn K. Bardet–Biedl syndrome proteins are required for the localization of G protein-coupled receptors to primary cilia. Proc Natl Acad Sci U S A. 2008;105(11):4242–6. 10.1073/pnas.0711027105. 18334641 10.1073/pnas.0711027105 PMC2393805

[117] Nachury MV. The molecular machines that traffic signaling receptors into and out of cilia. Curr Opin Cell Biol. 2018;51:124–31. 10.1016/j.ceb.2018.03.004. (**PubMed PMID: 29579578; PubMed Central PMCID: PMC5949257.**). 29579578 10.1016/j.ceb.2018.03.004 PMC5949257

[118] Ye F, Nager AR, Nachury MV. BBSome trains remove activated GPCRs from cilia by enabling passage through the transition zone. J Cell Biol. 2018;217(5):1847–68. 10.1083/jcb.201709041. (**PubMed PMID: 29483145; PubMed Central PMCID: PMC5940304.**). 29483145 10.1083/jcb.201709041 PMC5940304

[119] Shinde SR, Nager AR, Nachury MV. Ubiquitin chains earmark GPCRs for BBSome-mediated removal from cilia. J Cell Biol. 2020;219(12):e202003020. 10.1083/jcb.202003020. (**PubMed PMID: 33185668; PubMed Central PMCID: PMC7716378.**). 33185668 10.1083/jcb.202003020 PMC7716378

[120] Fujii T, Murai N, Aso S, Takatsu H, Shin HW, Katoh Y, et al. β-Arrestin mediates the export of ciliary GPR161 but not Smoothened together with the BBSome and intraflagellar transport machinery. J Cell Sci. 2025;138(20):jcs263793. 10.1242/jcs.263793. 40384633 10.1242/jcs.263793

[121] Nager AR, Goldstein JS, Herranz-Pérez V, Portran D, Ye F, Garcia-Verdugo JM, et al. An Actin network dispatches ciliary GPCRs into extracellular vesicles to modulate signaling. Cell. 2017;168(1–2):252-263.e14. 10.1016/j.cell.2016.11.036. (**PubMed PMID: 28017328; PubMed Central PMCID: PMC5235987.**). 28017328 10.1016/j.cell.2016.11.036 PMC5235987

[122] Garcia-Gonzalo FR, Corbit KC, Sirerol-Piquer MS, Ramaswami G, Otto EA, Noriega TR, et al. A transition zone complex regulates mammalian ciliogenesis and ciliary membrane composition. Nat Genet. 2011;43(8):776–84. 10.1038/ng.891. 21725307 10.1038/ng.891 PMC3145011

[123] Shimada H, Lu Q, Insinna-Kettenhofen C, Nagashima K, English MA, Semler EM, et al. In vitro modeling using ciliopathy-patient-derived cells reveals distinct cilia dysfunctions caused by CEP290 mutations. Cell Rep. 2017;20(2):384–96. 10.1016/j.celrep.2017.06.045. 28700940 10.1016/j.celrep.2017.06.045 PMC5553702

[124] Muñoz-Estrada J, Ferland RJ. Ahi1 promotes Arl13b ciliary recruitment, regulates Arl13b stability and is required for normal cell migration. J Cell Sci. 2019;132(17):jcs230680. 10.1242/jcs.230680. 31391239 10.1242/jcs.230680 PMC6771145

[125] Hu L, Wang B, Zhang Y. Serotonin 5-HT6 receptors affect cognition in a mouse model of Alzheimer’s disease by regulating cilia function. Alzheimers Res Ther. 2017;9(1):76. 10.1186/s13195-017-0304-4. 28931427 10.1186/s13195-017-0304-4 PMC5607612

[126] Su S, Phua SC, DeRose R, Chiba S, Narita K, Kalugin PN, et al. Genetically encoded calcium indicator illuminates calcium dynamics in primary cilia. Nat Methods. 2013;10(11):1105–7. 10.1038/nmeth.2647. 24056873 10.1038/nmeth.2647 PMC3860264

[127] Guadiana SM, Semple-Rowland S, Daroszewski D, Madorsky I, Breunig JJ, Mykytyn K, et al. Arborization of dendrites by developing neocortical neurons is dependent on primary cilia and Type 3 adenylyl cyclase. J Neurosci. 2013;33(6):2626–38. 10.1523/JNEUROSCI.2906-12.2013. (**PubMed PMID: 23392690.**). 23392690 10.1523/JNEUROSCI.2906-12.2013 PMC6619186

[128] Alhassen W, Kobayashi Y, Su J, Robbins B, Nguyen H, Myint T, et al. Regulation of brain primary cilia length by MCH signaling: Evidence from pharmacological, genetic, optogenetic, and chemogenic manipulations. Mol Neurobiol. 2022;59(1):245–65. 10.1007/s12035-021-02511-w. (**PubMed PMID: 34665407; PubMed Central PMCID: PMC9083846.**). 34665407 10.1007/s12035-021-02511-w PMC9083846

[129] Besschetnova TY, Kolpakova-Hart E, Guan Y, Zhou J, Olsen BR, Shah JV. Identification of signaling pathways regulating primary cilium length and flow-mediated adaptation. Curr Biol. 2010;20(2):182–7. 10.1016/j.cub.2009.11.072. 20096584 10.1016/j.cub.2009.11.072 PMC2990526

[130] Tu HQ, Li S, Xu YL, Zhang YC, Li PY, Liang LY, et al. Rhythmic cilia changes support SCN neuron coherence in circadian clock. Science. 2023;380(6648):972–9. 10.1126/science.abm1962. 37262147 10.1126/science.abm1962

[131] Mure LS, Le HD, Benegiamo G, Chang MW, Rios L, Jillani N, et al. Diurnal transcriptome atlas of a primate across major neural and peripheral tissues. Science. 2018;359(6381):eaao0318. 10.1126/science.aao0318. (**PubMed PMID: 29439024; PubMed Central PMCID: PMC5924732.**). 29439024 10.1126/science.aao0318 PMC5924732

[132] Korobeynikov V, Deneka AY, Golemis EA. Mechanisms for nonmitotic activation of Aurora-A at cilia. Biochem Soc Trans. 2017;45(1):37–49. 10.1042/BST20160142. 28202658 10.1042/BST20160142 PMC5860652

[133] Smith CEL, Lake AVR, Johnson CA. Primary cilia, ciliogenesis and the actin cytoskeleton: a little less resorption, a little more actin please. Front Cell Dev Biol. 2020;8:622822. 10.3389/fcell.2020.622822. 33392209 10.3389/fcell.2020.622822 PMC7773788

[134] Seo J, Kim J. Regulation of Hippo signaling by actin remodeling. BMB Rep. 2018;51(3):151–6. 10.5483/BMBRep.2018.51.3.012. 29353600 10.5483/BMBRep.2018.51.3.012 PMC5882222

[135] Kim J, Jo H, Hong H, Kim MH, Kim JM, Lee JK, et al. Actin remodelling factors control ciliogenesis by regulating YAP/TAZ activity and vesicle trafficking. Nat Commun. 2015;6(1):6781. 10.1038/ncomms7781. 25849865 10.1038/ncomms7781

[136] Rosengren T, Larsen LJ, Pedersen LB, Christensen ST, Møller LB. TSC1 and TSC2 regulate cilia length and canonical Hedgehog signaling via different mechanisms. Cell Mol Life Sci. 2018;75(14):2663–80. 10.1007/s00018-018-2761-8. 29396625 10.1007/s00018-018-2761-8 PMC6003990

[137] Takahashi K, Nagai T, Chiba S, Nakayama K, Mizuno K. Glucose deprivation induces primary cilium formation through mTORC1 inactivation. J Cell Sci. 2018;131(1):jcs208769. 10.1242/jcs.208769. 29180513 10.1242/jcs.208769

[138] Yasuhiro Y, Noboru M. Autophagy and Ciliogenesis. JMA J. 2024;4(3):207–15. 10.31662/jmaj.2021-0090. 10.31662/jmaj.2021-0090 PMC8355725 34414314

[139] Orhon I, Dupont N, Pampliega O, Cuervo AM, Codogno P. Autophagy and regulation of cilia function and assembly. Cell Death Differ. 2015;22(3):389–97. 10.1038/cdd.2014.171. 25361082 10.1038/cdd.2014.171 PMC4326575

[140] Pampliega O, Orhon I, Patel B, Sridhar S, Díaz-Carretero A, Beau I, et al. Functional interaction between autophagy and ciliogenesis. Nature. 2013;502(7470):194–200. 10.1038/nature12639. 24089209 10.1038/nature12639 PMC3896125

[141] Ávalos Y, Peña-Oyarzun D, Budini M, Morselli E, Criollo A. New roles of the primary cilium in autophagy. BioMed Res Int. 2017. 10.1155/2017/4367019. 28913352 10.1155/2017/4367019 PMC5587941

[142] Single-molecule imaging in the primary cilium. In: Methods in Cell Biology [Internet]. Elsevier; 2023 [cited 2026 Apr 25]. p. 59–83. https://linkinghub.elsevier.com/retrieve/pii/S0091679X2300003110.1016/bs.mcb.2023.01.003 10.1016/bs.mcb.2023.01.003 PMC10509820 37164543

[143] Ye F, Breslow DK, Koslover EF, Spakowitz AJ, Nelson WJ, Nachury MV. Single molecule imaging reveals a major role for diffusion in the exploration of ciliary space by signaling receptors. Elife. 2013;2:e00654. 10.7554/eLife.00654. 23930224 10.7554/eLife.00654 PMC3736543

[144] Prasai A, Ivashchenko O, Maskova K, Bykova S, Schmidt Cernohorska M, Stepanek O, et al. BBSome-deficient cells activate intraciliary CDC42 to trigger actin-dependent ciliary ectocytosis. EMBO Rep. 2025;26(1):36–60. 10.1038/s44319-024-00326-z. (**PubMedPMID:39587330;PubMedCentralPMCID:PMC11724091**). 39587330 10.1038/s44319-024-00326-z PMC11724091

[145] Hu HB, Song ZQ, Song GP, Li S, Tu HQ, Wu M, et al. LPA signaling acts as a cell-extrinsic mechanism to initiate cilia disassembly and promote neurogenesis. Nat Commun. 2021;12(1):662. 10.1038/s41467-021-20986-y. 33510165 10.1038/s41467-021-20986-y PMC7843646

[146] Hasenpusch-Theil K, Laclef C, Colligan M, Fitzgerald E, Howe K, Carroll E, et al. A transient role of the ciliary gene Inpp5e in controlling direct versus indirect neurogenesis in cortical development. Elife. 2020;9:e58162. 10.7554/eLife.58162. 32840212 10.7554/eLife.58162 PMC7481005

[147] Li A, Saito M, Chuang JZ, Tseng YY, Dedesma C, Tomizawa K, et al. Ciliary transition zone activation of phosphorylated Tctex-1 controls ciliary resorption, S-phase entry and fate of neural progenitors. Nat Cell Biol. 2011;13(4):402–11. 10.1038/ncb2218. (**PubMedPMID:21394082;PubMedCentralPMCID:PMC4018803**). 21394082 10.1038/ncb2218 PMC4018803

[148] Heurck RV, Bonnefont J, Wojno M, Suzuki IK, Velez-Bravo FD, Erkol E, et al. CROCCP2 acts as a human-specific modifier of cilia dynamics and mTOR signaling to promote expansion of cortical progenitors. Neuron. 2023;111(1):65-80.e6. 10.1016/j.neuron.2022.10.018. (**PubMed PMID: 36334595**). 36334595 10.1016/j.neuron.2022.10.018 PMC9831670

[149] Takeo Y, Kurabayashi N, Nguyen MD, Sanada K. The G protein-coupled receptor GPR157 regulates neuronal differentiation of radial glial progenitors through the Gq-IP3 pathway. Sci Rep. 2016;6(1):25180. 10.1038/srep25180. 27142930 10.1038/srep25180 PMC4855140

[150] Chang CH, Zanini M, Shirvani H, Cheng JS, Yu H, Feng CH, et al. Atoh1 controls primary cilia formation to allow for SHH-triggered granule neuron progenitor proliferation. Dev Cell. 2019;48(2):184-199.e5. 10.1016/j.devcel.2018.12.017. (**PubMed PMID: 30695697**). 30695697 10.1016/j.devcel.2018.12.017

[151] Lin IH, Li YR, Chang CH, Cheng YW, Wang YT, Tsai YS, et al. Regulation of primary cilia disassembly through HUWE1-mediated TTBK2 degradation plays a crucial role in cerebellar development and medulloblastoma growth. Cell Death Differ. 2024;31(10):1349–61. 10.1038/s41418-024-01325-2. 38879724 10.1038/s41418-024-01325-2 PMC11445238

[152] Han YG, Spassky N, Romaguera-Ros M, Garcia-Verdugo JM, Aguilar A, Schneider-Maunoury S, et al. Hedgehog signaling and primary cilia are required for the formation of adult neural stem cells. Nat Neurosci. 2008;11(3):277–84. 10.1038/nn2059. 18297065 10.1038/nn2059

[153] Breunig JJ, Sarkisian MR, Arellano JI, Morozov YM, Ayoub AE, Sojitra S, et al. Primary cilia regulate hippocampal neurogenesis by mediating sonic hedgehog signaling. Proc Natl Acad Sci. 2008;105(35):13127–32. 10.1073/pnas.0804558105. 18728187 10.1073/pnas.0804558105 PMC2529104

[154] Bashford AL, Subramanian V. Hippocampals neurogenesis is impaired in mice with a deletion in the coiled coil domain of Talpid3-implications for Joubert syndrome. Hum Mol Genet. 2022;31(19):3245–65. 10.1093/hmg/ddac095. (**PubMedPMID:35470378;PubMedCentralPMCID:PMC9523558**). 35470378 10.1093/hmg/ddac095 PMC9523558

[155] Kong JH, Yang L, Dessaud E, Chuang K, Moore DM, Rohatgi R, et al. Notch activity modulates the responsiveness of neural progenitors to sonic hedgehog signaling. Dev Cell. 2015;33(4):373–87. 10.1016/j.devcel.2015.03.005. (**PubMedPMID:25936505;PubMedCentralPMCID:PMC4449290**). 25936505 10.1016/j.devcel.2015.03.005 PMC4449290

[156] Andreu-Cervera A, Anselme I, Karam A, Laclef C, Catala M, Schneider-Maunoury S. The ciliopathy gene FTM/RPGRIP1L controls mouse forebrain patterning via region-specific modulation of hedgehog/gli signaling. J Neurosci. 2019;39(13):2398–415. 10.1523/JNEUROSCI.2199-18.2019. (**PubMedPMID:30692221;PubMedCentralPMCID:PMC6435827**). 30692221 10.1523/JNEUROSCI.2199-18.2019 PMC6435827

[157] Besse L, Neti M, Anselme I, Gerhardt C, Rüther U, Laclef C, et al. Primary cilia control telencephalic patterning and morphogenesis via Gli3 proteolytic processing. Development. 2011;138(10):2079–88. 10.1242/dev.059808. (**PubMed PMID: 21490064**). 21490064 10.1242/dev.059808

[158] Jang J, Wang Y, Lalli MA, Guzman E, Godshalk SE, Zhou H, et al. Primary cilium-autophagy-Nrf2 (PAN) axis activation commits human embryonic stem cells to a neuroectoderm fate. Cell. 2016;165(2):410–20. 10.1016/j.cell.2016.02.014. (**PubMed PMID: 27020754**). 27020754 10.1016/j.cell.2016.02.014

[159] Higginbotham H, Guo J, Yokota Y, Umberger NL, Su CY, Li J, et al. Arl13b-regulated activities of primary cilia are essential for the formation of the polarized radial glial scaffold. Nat Neurosci. 2013;16(8):1000–7. 10.1038/nn.3451. (**PubMedPMID:23817546;PubMedCentralPMCID:PMC3866024**). 23817546 10.1038/nn.3451 PMC3866024

[160] Guo J, Higginbotham H, Li J, Nichols J, Hirt J, Ghukasyan V, et al. Developmental disruptions underlying brain abnormalities in ciliopathies. Nat Commun. 2015;6(1):7857. 10.1038/ncomms8857. 26206566 10.1038/ncomms8857 PMC4515781

[161] Baudoin JP, Viou L, Launay PS, Luccardini C, Espeso Gil S, Kiyasova V, et al. Tangentially migrating neurons assemble a primary cilium that promotes their reorientation to the cortical plate. Neuron. 2012;76(6):1108–22. 10.1016/j.neuron.2012.10.027. 23259947 10.1016/j.neuron.2012.10.027

[162] Wang X, Tsai JW, Imai JH, Lian WN, Vallee RB, Shi SH. Asymmetric centrosome inheritance maintains neural progenitors in the neocortex. Nature. 2009;461(7266):947–55. 10.1038/nature08435. (**PubMed PMID: 19829375; PubMed Central PMCID: PMC2764320**). 19829375 10.1038/nature08435 PMC2764320

[163] Yang J, Mirhosseiniardakani S, Qiu L, Bicja K, Del Greco A, Lin KJ, et al. Cilia directionality reveals a slow reverse movement of principal neurons for positioning and lamina refinement in the cerebral cortex. Development. 2025;152(5):DEV204300. 10.1242/dev.204300. (**PubMed PMID: 40066717; PubMed Central PMCID: PMC12050088.**). 40066717 10.1242/dev.204300 PMC12050088

[164] Guo J, Otis JM, Higginbotham H, Monckton C, Cheng J, Asokan A, et al. Primary cilia signaling shapes the development of interneuronal connectivity. Dev Cell. 2017;42(3):286-300.e4. 10.1016/j.devcel.2017.07.010. (**PubMed PMID: 28787594**). 28787594 10.1016/j.devcel.2017.07.010 PMC5571900

[165] Guo J, Otis JM, Suciu SK, Catalano C, Xing L, Constable S, et al. Primary cilia signaling promotes axonal tract development and is disrupted in Joubert syndrome-related disorders models. Dev Cell. 2019;51(6):759-774.e5. 10.1016/j.devcel.2019.11.005. (**PubMed PMID: 31846650**). 31846650 10.1016/j.devcel.2019.11.005 PMC6953258

[166] Ferent J, Constable S, Gigante ED, Yam PT, Mariani LE, Legué E, et al. The ciliary protein Arl13b functions outside of the primary cilium in Shh-mediated axon guidance. Cell Rep. 2019;29(11):3356-3366.e3. 10.1016/j.celrep.2019.11.015. 31825820 10.1016/j.celrep.2019.11.015 PMC6927553

[167] Magnani D, Morlé L, Hasenpusch-Theil K, Paschaki M, Jacoby M, Schurmans S, et al. The ciliogenic transcription factor Rfx3 is required for the formation of the thalamocortical tract by regulating the patterning of prethalamus and ventral telencephalon. Hum Mol Genet. 2015;24(9):2578–93. 10.1093/hmg/ddv021. (**PubMed PMID: 25631876**). 25631876 10.1093/hmg/ddv021

[168] Gupta R, Duff MC, Denburg NL, Cohen NJ, Bechara A, Tranel D. Declarative memory is critical for sustained advantageous complex decision-making. Neuropsychologia. 2009;47(7):1686–93. 10.1016/j.neuropsychologia.2009.02.007. (**PubMed PMID: 19397863; PubMed Central PMCID: PMC2697903**). 19397863 10.1016/j.neuropsychologia.2009.02.007 PMC2697903

[169] Poretti A, Huisman TAGM, Scheer I, Boltshauser E. Joubert syndrome and related disorders: spectrum of neuroimaging findings in 75 patients. AJNR Am J Neuroradiol. 2011;32(8):1459–63. 10.3174/ajnr.A2517. (**PubMed PMID: 21680654; PubMed Central PMCID: PMC7964342**). 21680654 10.3174/ajnr.A2517 PMC7964342

[170] Verrotti A, Spalice A, Ursitti F, Papetti L, Mariani R, Castronovo A, et al. New trends in neuronal migration disorders. Eur J Paediatr Neurol. 2010;14(1):1–12. 10.1016/j.ejpn.2009.01.005. 19264520 10.1016/j.ejpn.2009.01.005

[171] Muraki K, Tanigaki K. Neuronal migration abnormalities and its possible implications for schizophrenia. Front Neurosci. 2015. 10.3389/fnins.2015.00074. 25805966 10.3389/fnins.2015.00074 PMC4354421

[172] Feng G, Wang Y, Huang W, Chen H, Cheng J, Shu N. Spatial and temporal pattern of structure–function coupling of human brain connectome with development. Elife. 2024;13:RP93325. 10.7554/eLife.93325. 38900563 10.7554/eLife.93325 PMC11189631

[173] Pang JC, Aquino KM, Oldehinkel M, Robinson PA, Fulcher BD, Breakspear M, et al. Geometric constraints on human brain function. Nature. 2023;618(7965):566–74. 10.1038/s41586-023-06098-1. 37258669 10.1038/s41586-023-06098-1 PMC10266981

[174] Bullmore E, Sporns O. The economy of brain network organization. Nat Rev Neurosci. 2012;13(5):336–49. 10.1038/nrn3214. 22498897 10.1038/nrn3214

[175] Bertolero MA, Yeo BTT, D’Esposito M. The modular and integrative functional architecture of the human brain. Proc Natl Acad Sci. 2015. 10.1073/pnas.1510619112. 26598686 10.1073/pnas.1510619112 PMC4679040

[176] Ozeki H, Finn IM, Schaffer ES, Miller KD, Ferster D. Inhibitory stabilization of the cortical network underlies visual surround suppression. Neuron. 2009;62(4):578–92. 10.1016/j.neuron.2009.03.028. (**PubMed PMID: 19477158; PubMed Central PMCID: PMC2691725**). 19477158 10.1016/j.neuron.2009.03.028 PMC2691725

[177] Winnubst J, Bas E, Ferreira TA, Wu Z, Economo MN, Edson P, et al. Reconstruction of 1,000 projection neurons reveals new cell types and organization of long-range connectivity in the mouse brain. Cell. 2019;179(1):268-281.e13. 10.1016/j.cell.2019.07.042. (**PubMed PMID: 31495573; PubMed Central PMCID: PMC6754285**). 31495573 10.1016/j.cell.2019.07.042 PMC6754285

[178] Amador-Arjona A, Elliott J, Miller A, Ginbey A, Pazour GJ, Enikolopov G, et al. Primary cilia regulate proliferation of amplifying progenitors in adult hippocampus: implications for learning and memory. J Neurosci. 2011;31(27):9933–44. 10.1523/JNEUROSCI.1062-11.2011. 21734285 10.1523/JNEUROSCI.1062-11.2011 PMC3758574

[179] Dupuy V, Prieur M, Pizzoccaro A, Margarido C, Valjent E, Bockaert J, et al. Spatiotemporal dynamics of 5-HT6 receptor ciliary localization during mouse brain development. Neurobiol Dis. 2023;176:105949. 10.1016/j.nbd.2022.105949. 36496200 10.1016/j.nbd.2022.105949

[180] Stubbs T, Koemeter-Cox A, Bingman JI, Zhao F, Kalyanasundaram A, Rowland LA, et al. Disruption of dopamine receptor 1 localization to primary cilia impairs signaling in striatal neurons. J Neurosci. 2022;42(35):6692–705. 10.1523/JNEUROSCI.0497-22.2022. (**PubMed PMID: 35882560.**). 35882560 10.1523/JNEUROSCI.0497-22.2022 PMC9436016

[181] Diniz GB, Battagello DS, Klein MO, Bono BSM, Ferreira JGP, Motta‐Teixeira LC, et al. Ciliary melanin‐concentrating hormone receptor 1 (MCHR1) is widely distributed in the murine CNS in a sex‐independent manner. J Neurosci Res. 2020;98(10):2045–71. 10.1002/jnr.24651. 32530066 10.1002/jnr.24651

[182] Siljee JE, Wang Y, Bernard AA, Ersoy BA, Zhang S, Marley A, et al. Subcellular localization of MC4R with ADCY3 at neuronal primary cilia underlies a common pathway for genetic predisposition to obesity. Nat Genet. 2018;50(2):180–5. 10.1038/s41588-017-0020-9. (**PubMed PMID: 29311635; PubMed Central PMCID: PMC5805646.**). 29311635 10.1038/s41588-017-0020-9 PMC5805646

[183] Loktev AV, Jackson PK. Neuropeptide Y family receptors traffic via the Bardet-Biedl syndrome pathway to signal in neuronal primary cilia. Cell Rep. 2013;5(5):1316–29. 10.1016/j.celrep.2013.11.011. (**PubMed PMID: 24316073.**). 24316073 10.1016/j.celrep.2013.11.011

[184] Brailov I, Bancila M, Brisorgueil MJ, Miquel MC, Hamon M, Vergé D. Localization of 5-HT6 receptors at the plasma membrane of neuronal cilia in the rat brain. Brain Res. 2000;872(1):271–5. 10.1016/S0006-8993(00)02519-1. 10924708 10.1016/s0006-8993(00)02519-1

[185] Brodsky M, Lesiak AJ, Croicu A, Cohenca N, Sullivan JM, Neumaier JF. 5-HT6 receptor blockade regulates primary cilia morphology in striatal neurons. Brain Res. 2017;1660:10–9. 10.1016/j.brainres.2017.01.010. 28087224 10.1016/j.brainres.2017.01.010 PMC5392252

[186] Gérard C, Martres MP, Lefèvre K, Miquel MC, Vergé D, Lanfumey L, et al. Immuno-localization of serotonin 5-HT6 receptor-like material in the rat central nervous system. Brain Res. 1997;746(1):207–19. 10.1016/S0006-8993(96)01224-3. 9037500 10.1016/s0006-8993(96)01224-3

[187] Nirogi R, Ieni J, Goyal VK, Ravula J, Jetta S, Shinde A, et al. Effect of Masupirdine (SUVN‐502) on cognition in patients with moderate Alzheimer’s disease: a randomized, double‐blind, phase 2, proof‐of‐concept study. Alzheimers Dement N Y. 2022;8(1):e12307. 10.1002/trc2.12307. (**PubMed PMID: 35662833; PubMed Central PMCID: PMC9157584.**). 35662833 10.1002/trc2.12307 PMC9157584

[188] Wilkinson D, Windfeld K, Colding-Jørgensen E. Safety and efficacy of Idalopirdine, a 5-HT6 receptor antagonist, in patients with moderate Alzheimer’s disease (LADDER): a randomised, double-blind, placebo-controlled phase 2 trial. Lancet Neurol. 2014;13(11):1092–9. 10.1016/S1474-4422(14)70198-X. (**PubMed PMID: 25297016.**). 25297016 10.1016/S1474-4422(14)70198-X

[189] Frölich L, Atri A, Ballard C, Tariot PN, Molinuevo JL, Boneva N, et al. Open-label, multicenter, Phase III extension study of Idalopirdine as adjunctive to Donepezil for the treatment of mild-moderate Alzheimer’s disease. J Alzheimers Dis. 2019;67(1):303–13. 10.3233/JAD-180595. 30636738 10.3233/JAD-180595

[190] Morozova M, Burminskiy D, Rupchev G, Lepilkina T, Potanin S, Beniashvili A, et al. 5-HT6 receptor antagonist as an adjunct treatment targeting residual symptoms in patients with Schizophrenia: unexpected sex-related effects (double-blind placebo-controlled trial). J Clin Psychopharmacol. 2017;37(2):169. 10.1097/JCP.0000000000000673. 28141622 10.1097/JCP.0000000000000673

[191] Foley AG, Murphy KJ, Hirst WD, Gallagher HC, Hagan JJ, Upton N, et al. The 5-HT6 receptor antagonist SB-271046 reverses Scopolamine-disrupted consolidation of a passive avoidance task and ameliorates spatial task deficits in aged rats. Neuropsychopharmacology. 2004;29(1):93–100. 10.1038/sj.npp.1300332. 14571256 10.1038/sj.npp.1300332

[192] Stean TO, Hirst WD, Thomas DR, Price GW, Rogers D, Riley G, et al. Pharmacological profile of SB-357134: a potent, selective, brain penetrant, and orally active 5-HT(6) receptor antagonist. Pharmacol Biochem Behav. 2002;71(4):645–54. 10.1016/s0091-3057(01)00742-0. (**PubMed PMID: 11888556.**). 11888556 10.1016/s0091-3057(01)00742-0

[193] Woolley ML, Bentley JC, Sleight AJ, Marsden CA, Fone KCF. A role for 5-ht6 receptors in retention of spatial learning in the Morris water maze. Neuropharmacology. 2001;41(2):210–9. 10.1016/S0028-3908(01)00056-9. 11489457 10.1016/s0028-3908(01)00056-9

[194] King MV, Sleight AJ, Woolley ML, Topham IA, Marsden CA, Fone KCF. 5-HT6 receptor antagonists reverse delay-dependent deficits in novel object discrimination by enhancing consolidation—an effect sensitive to NMDA receptor antagonism. Neuropharmacology. 2004;47(2):195–204. 10.1016/j.neuropharm.2004.03.012. 15223298 10.1016/j.neuropharm.2004.03.012

[195] Pereira M, Martynhak BJ, Andreatini R, Svenningsson P. 5-HT6 receptor agonism facilitates emotional learning. Front Pharmacol. 2015;6:200. 10.3389/fphar.2015.00200. (**PubMed PMID: 26441657; PubMed Central PMCID: PMC4584947.**). 26441657 10.3389/fphar.2015.00200 PMC4584947

[196] Kendall I, Slotten HA, Codony X, Burgueño J, Pauwels PJ, Vela JM, et al. E-6801, a 5-HT6 receptor agonist, improves recognition memory by combined modulation of cholinergic and glutamatergic neurotransmission in the rat. Psychopharmacology. 2011;213(2):413–30. 10.1007/s00213-010-1854-3. 20405281 10.1007/s00213-010-1854-3

[197] Amodeo DA, Peterson S, Pahua A, Posadas R, Hernandez A, Hefner E, et al. 5-HT6 receptor agonist EMD386088 impairs behavioral flexibility and working memory. Behav Brain Res. 2018;349:8–15. 10.1016/j.bbr.2018.04.032. 29715539 10.1016/j.bbr.2018.04.032

[198] Meneses A, Perez-Garcia G, Liy-Salmeron G, Flores-Galvez D, Castillo C, Castillo E. The effects of the 5-HT6 receptor agonist EMD and the 5-HT7 receptor agonist AS19 on memory formation. Behav Brain Res. 2008;195(1):112–9. 10.1016/j.bbr.2007.11.023. 18191236 10.1016/j.bbr.2007.11.023

[199] Brouard JT, Schweimer JV, Houlton R, Burnham KE, Quérée P, Sharp T. Pharmacological evidence for 5-HT6 receptor modulation of 5-HT neuron firing in vivo. ACS Chem Neurosci. 2015;6(7):1241–7. 10.1021/acschemneuro.5b00061. 25837696 10.1021/acschemneuro.5b00061

[200] Hirst WD, Stean TO, Rogers DC, Sunter D, Pugh P, Moss SF, et al. SB-399885 is a potent, selective 5-HT6 receptor antagonist with cognitive enhancing properties in aged rat water maze and novel object recognition models. Eur J Pharmacol. 2006;553(1):109–19. 10.1016/j.ejphar.2006.09.049. 17069795 10.1016/j.ejphar.2006.09.049

[201] Zhang MY, Hughes ZA, Kerns EH, Lin Q, Beyer CE. Development of a liquid chromatography/tandem mass spectrometry method for the quantitation of acetylcholine and related neurotransmitters in brain microdialysis samples. J Pharm Biomed Anal. 2007;44(2):586–93. 10.1016/j.jpba.2007.02.024. (**Hyphenated Techniques in Pharmaceutical and Biomedical Analysis 2006**). 17383138 10.1016/j.jpba.2007.02.024

[202] Schechter LE, Lin Q, Smith DL, Zhang G, Shan Q, Platt B, et al. Neuropharmacological profile of novel and selective 5-HT6 receptor agonists: WAY-181187 and WAY-208466. Neuropsychopharmacology. 2008;33(6):1323–35. 10.1038/sj.npp.1301503. 17625499 10.1038/sj.npp.1301503

[203] Tassone A, Madeo G, Schirinzi T, Vita D, Puglisi F, Ponterio G, et al. Activation of 5-HT6 receptors inhibits corticostriatal glutamatergic transmission. Neuropharmacology. 2011;61(4):632–7. 10.1016/j.neuropharm.2011.05.004. 21619890 10.1016/j.neuropharm.2011.05.004

[204] Wang HY, Lu CW, Lin TY, Kuo JR, Wang SJ. WAY208466 inhibits glutamate release at hippocampal nerve terminals. Eur J Pharmacol. 2016;781:117–27. 10.1016/j.ejphar.2016.04.010. 27068148 10.1016/j.ejphar.2016.04.010

[205] Mørk A, Russell RV, de Jong IEM, Smagin G. Effects of the 5-HT6 receptor antagonist idalopirdine on extracellular levels of monoamines, glutamate and acetylcholine in the rat medial prefrontal cortex. Eur J Pharmacol. 2017;799:1–6. 10.1016/j.ejphar.2017.02.010. 28188762 10.1016/j.ejphar.2017.02.010

[206] Lacroix LP, Dawson LA, Hagan JJ, Heidbreder CA. 5-HT6 receptor antagonist SB-271046 enhances extracellular levels of monoamines in the rat medial prefrontal cortex. Synapse. 2004;51(2):158–64. 10.1002/syn.10288. 14618683 10.1002/syn.10288

[207] Wang B, Hu L, Sun Z, Zhang Y. Cilia function is associated with axon initial segment morphology. Biochem Biophys Res Commun. 2019;516(1):15–21. 10.1016/j.bbrc.2019.05.172. 31186137 10.1016/j.bbrc.2019.05.172

[208] Wang H, Li Y, Li X, Sun Z, Yu F, Pashang A, et al. The primary cilia are associated with the axon initial segment in neurons. Adv Sci. 2025;12(9):2407405. 10.1002/advs.202407405. 10.1002/advs.202407405 PMC11884599 39804991

[209] Chand AN, Galliano E, Chesters RA, Grubb MS. A distinct subtype of dopaminergic interneuron displays inverted structural plasticity at the axon initial segment. J Neurosci. 2015;35(4):1573–90. 10.1523/JNEUROSCI.3515-14.2015. (**PubMed PMID: 25632134; PubMed Central PMCID: PMC4308603.**). 25632134 10.1523/JNEUROSCI.3515-14.2015 PMC4308603

[210] Gulledge AT, Bravo JJ. Neuron morphology influences axon initial segment plasticity. eNeuro. 2016;3(1):ENEURO.0085-15.2016. 10.1523/ENEURO.0085-15.2016. (**PubMed PMID: 27022619; PubMed Central PMCID: PMC4756267**). 27022619 10.1523/ENEURO.0085-15.2016 PMC4756267

[211] Einstein EB, Patterson CA, Hon BJ, Regan KA, Reddi J, Melnikoff DE, et al. Somatostatin signaling in neuronal cilia is criticalfor object recognition memory. J Neurosci. 2010;30(12):4306–14. 10.1523/JNEUROSCI.5295-09.2010. (**PubMed PMID: 20335466.**). 20335466 10.1523/JNEUROSCI.5295-09.2010 PMC3842454

[212] Tereshko L, Gao Y, Cary BA, Turrigiano GG, Sengupta P. Ciliary neuropeptidergic signaling dynamically regulates excitatory synapses in postnatal neocortical pyramidal neurons. Elife. 2021;10:e65427. 10.7554/eLife.65427. (**PubMed PMID: 33650969; PubMed Central PMCID: PMC7952091**). 33650969 10.7554/eLife.65427 PMC7952091

[213] Isaacson JS, Scanziani M. How inhibition shapes cortical activity. Neuron. 2011;72(2):231–43. 10.1016/j.neuron.2011.09.027. 22017986 10.1016/j.neuron.2011.09.027 PMC3236361

[214] Lewis DA, Curley AA, Glausier JR, Volk DW. Cortical parvalbumin interneurons and cognitive dysfunction in schizophrenia. Trends Neurosci. 2012;35(1):57–67. 10.1016/j.tins.2011.10.004. (**PubMed PMID: 22154068.**). 22154068 10.1016/j.tins.2011.10.004 PMC3253230

[215] Etherton MR, Tabuchi K, Sharma M, Ko J, Südhof TC. An autism‐associated point mutation in the neuroligin cytoplasmic tail selectively impairs AMPA receptor‐mediated synaptic transmission in hippocampus. EMBO J. 2011;30(14):2908–19. 10.1038/emboj.2011.182. 21642956 10.1038/emboj.2011.182 PMC3160244

[216] Bateup HS, Johnson CA, Denefrio CL, Saulnier JL, Kornacker K, Sabatini BL. Excitatory/inhibitory synaptic imbalance leads to hippocampal hyperexcitability in mouse models of Tuberous Sclerosis. Neuron. 2013;78(3):510–22. 10.1016/j.neuron.2013.03.017. 23664616 10.1016/j.neuron.2013.03.017 PMC3690324

[217] Barberis A, Petrini EM, Mozrzymas JW. Impact of synaptic neurotransmitter concentration time course on the kinetics and pharmacological modulation of inhibitory synaptic currents. Front Cell Neurosci. 2011;5:6. 10.3389/fncel.2011.00006. (**PubMed PMID: 21734864; PubMed Central PMCID: PMC3123770.**). 21734864 10.3389/fncel.2011.00006 PMC3123770

[218] Phua SC, Tan YL, Kok AMY, Senol E, Chiam CJH, Lee CY, et al. A distinct parabrachial–to–lateral hypothalamus circuit for motivational suppression of feeding by nociception. Sci Adv. 2021;7(19):eabe4323. 10.1126/sciadv.abe4323. 33962958 10.1126/sciadv.abe4323 PMC8104871

[219] Li MM, Madara JC, Steger JS, Krashes MJ, Balthasar N, Campbell JN, et al. The paraventricular hypothalamus regulates satiety and prevents obesity via two genetically distinct circuits. Neuron. 2019;102(3):653-667.e6. 10.1016/j.neuron.2019.02.028. (**PubMed PMID: 30879785; PubMed Central PMCID: PMC6508999.**). 30879785 10.1016/j.neuron.2019.02.028 PMC6508999

[220] Shah BP, Vong L, Olson DP, Koda S, Krashes MJ, Ye C, et al. MC4R-expressing glutamatergic neurons in the paraventricular hypothalamus regulate feeding and are synaptically connected to the parabrachial nucleus. Proc Natl Acad Sci U S A. 2014;111(36):13193–8. 10.1073/pnas.1407843111. 25157144 10.1073/pnas.1407843111 PMC4246954

[221] Xu Y, Wu Z, Sun H, Zhu Y, Kim ER, Lowell BB, et al. Glutamate mediates the function of melanocortin receptors 4 on Sim1 neurons in body weight regulation. Cell Metab. 2013;18(6):10.1016/j.cmet.2013.11.003. 10.1016/j.cmet.2013.11.003. (**PubMed PMID: 24315371; PubMed Central PMCID: PMC3880549**). 10.1016/j.cmet.2013.11.003 PMC3880549 24315371

[222] Fenselau H, Campbell JN, Verstegen AMJ, Madara JC, Xu J, Shah BP, et al. A rapidly acting glutamatergic ARC→PVH satiety circuit postsynaptically regulated by α-MSH. Nat Neurosci. 2017;20(1):42–51. 10.1038/nn.4442. (**PubMed PMID: 27869800; PubMed Central PMCID: PMC5191921.**). 27869800 10.1038/nn.4442 PMC5191921

[223] Deem JD, Faber CL, Morton GJ. AgRP neurons: regulators of feeding, energy expenditure, and behavior. FEBS J. 2022;289(8):2362–81. 10.1111/febs.16176. (**PubMed PMID: 34469623; PubMed Central PMCID: PMC9040143.**). 34469623 10.1111/febs.16176 PMC9040143

[224] Biglari N, Gaziano I, Schumacher J, Radermacher J, Paeger L, Klemm P, et al. Functionally distinct POMC-expressing neuron subpopulations in hypothalamus revealed by intersectional targeting. Nat Neurosci. 2021;24(7):913–29. 10.1038/s41593-021-00854-0. 34002087 10.1038/s41593-021-00854-0 PMC8249241

[225] Trotta M, Bello EP, Alsina R, Tavella MB, Ferrán JL, Rubinstein M, et al. Hypothalamic Pomc expression restricted to GABAergic neurons suppresses Npy overexpression and restores food intake in obese mice. Mol Metab. 2020;37:100985. 10.1016/j.molmet.2020.100985. (**PubMed PMID: 32311511; PubMed Central PMCID: PMC7292867.**). 32311511 10.1016/j.molmet.2020.100985 PMC7292867

[226] Farooqi IS, Keogh JM, Yeo GSH, Lank EJ, Cheetham T, O’Rahilly S. Clinical spectrum of obesity and mutations in the melanocortin 4 receptor gene. N Engl J Med. 2003;348(12):1085–95. 10.1056/NEJMoa022050. 12646665 10.1056/NEJMoa022050

[227] Loos RJF. The genetic epidemiology of *melanocortin 4 receptor* variants. Eur J Pharmacol. 2011;660(1):156–64. 10.1016/j.ejphar.2011.01.033. (**Special issue on Melanocortins**). 21295023 10.1016/j.ejphar.2011.01.033

[228] Zorn S, Bounds R, Williamson A, Lawler K, Hanssen R, Keogh J, et al. Obesity due to MC4R deficiency is associated with reduced cholesterol, triglycerides and cardiovascular disease risk. Nat Med. 2025. 10.1038/s41591-025-03976-1. 41102563 10.1038/s41591-025-03976-1 PMC12705457

[229] Glas E, Mückter H, Gudermann T, Breit A. Exchange factors directly activated by cAMP mediate melanocortin 4 receptor-induced gene expression. Sci Rep. 2016;6(1):32776. 10.1038/srep32776. 27612207 10.1038/srep32776 PMC5017209

[230] Tong T, Shen Y, Lee HW, Yu R, Park T. Adenylyl cyclase 3 haploinsufficiency confers susceptibility to diet-induced obesity and insulin resistance in mice. Sci Rep. 2016;6(1):34179. 10.1038/srep34179. 27678003 10.1038/srep34179 PMC5039768

[231] Garfield AS, Li C, Madara JC, Shah BP, Webber E, Steger JS, et al. A neural basis for melanocortin-4 receptor regulated appetite. Nat Neurosci. 2015;18(6):863–71. 10.1038/nn.4011. 25915476 10.1038/nn.4011 PMC4446192

[232] Antal-Zimanyi I, Khawaja X. The role of melanin-concentrating hormone in energy homeostasis and mood disorders. J Mol Neurosci MN. 2009;39(1–2):86–98. 10.1007/s12031-009-9207-6. 19418262 10.1007/s12031-009-9207-6

[233] Marley A, von Zastrow M. DISC1 regulates primary cilia that display specific dopamine receptors. PLoS ONE. 2010;5(5):e10902. 10.1371/journal.pone.0010902. 20531939 10.1371/journal.pone.0010902 PMC2878344

[234] Domire JS, Green JA, Lee KG, Johnson AD, Askwith CC, Mykytyn K. Dopamine receptor 1 localizes to neuronal cilia in a dynamic process that requires the Bardet-Biedl syndrome proteins. Cell Mol Life Sci. 2011;68(17):2951–60. 10.1007/s00018-010-0603-4. 21152952 10.1007/s00018-010-0603-4 PMC3368249

[235] Yao WD, Spealman RD, Zhang J. Dopaminergic signaling in dendritic spines. Biochem Pharmacol. 2008;75(11):2055–69. 10.1016/j.bcp.2008.01.018. 18353279 10.1016/j.bcp.2008.01.018 PMC2443745

[236] Scott L, Kruse MS, Forssberg H, Brismar H, Greengard P, Aperia A. Selective up-regulation of dopamine D1 receptors in dendritic spines by NMDA receptor activation. Proc Natl Acad Sci U S A. 2002;99(3):1661–4. 10.1073/pnas.032654599. 11818555 10.1073/pnas.032654599 PMC122247

[237] Hu JL, Liu G, Li YC, Gao WJ, Huang YQ. Dopamine D1 receptor-mediated NMDA receptor insertion depends on Fyn but not Src kinase pathway in prefrontal cortical neurons. Mol Brain. 2010;3(1):20. 10.1186/1756-6606-3-20. 20569495 10.1186/1756-6606-3-20 PMC2902469

[238] Dunah AW, Standaert DG. Dopamine D1 receptor-dependent trafficking of striatal NMDA glutamate receptors to the postsynaptic membrane. J Neurosci. 2001;21(15):5546–58. 10.1523/JNEUROSCI.21-15-05546.2001. (**PubMedPMID:11466426;PubMedCentralPMCID:PMC6762635**). 11466426 10.1523/JNEUROSCI.21-15-05546.2001 PMC6762635

[239] Gao C, Sun X, Wolf ME. Activation of D1 dopamine receptors increases surface expression of AMPA receptors and facilitates their synaptic incorporation in cultured hippocampal neurons. J Neurochem. 2006;98(5):1664–77. 10.1111/j.1471-4159.2006.03999.x. (**PubMed PMID: 16800848**). 16800848 10.1111/j.1471-4159.2006.03999.x

[240] Sun X, Zhao Y, Wolf ME. Dopamine receptor stimulation modulates AMPA receptor synaptic insertion in prefrontal cortex neurons. J Neurosci Off J Soc Neurosci. 2005;25(32):7342–51. 10.1523/JNEUROSCI.4603-04.2005. (**PubMedPMID:16093384;PubMedCentralPMCID:PMC6725299**). 10.1523/JNEUROSCI.4603-04.2005 PMC6725299 16093384

[241] Hallett PJ, Spoelgen R, Hyman BT, Standaert DG, Dunah AW. Dopamine D1 activation potentiates striatal NMDA receptors by tyrosine phosphorylation-dependent subunit trafficking. J Neurosci. 2006;26(17):4690–700. 10.1523/JNEUROSCI.0792-06.2006. (**PubMed PMID: 16641250**). 16641250 10.1523/JNEUROSCI.0792-06.2006 PMC6674081

[242] Suarez LM, Solis O, Sanz-Magro A, Alberquilla S, Moratalla R. Dopamine D1 receptors regulate spines in striatal direct-pathway and indirect-pathway neurons. Mov Disord Off J Mov Disord Soc. 2020;35(10):1810–21. 10.1002/mds.28174. (**PubMed PMID: 32643147**). 10.1002/mds.28174 32643147

[243] del Puerto A, Díaz-Hernández JI, Tapia M, Gomez-Villafuertes R, Benitez MJ, Zhang J, et al. Adenylate cyclase 5 coordinates the action of ADP, P2Y1, P2Y13 and ATP-gated P2X7 receptors on axonal elongation. J Cell Sci. 2012;125(1):176–88. 10.1242/jcs.091736. 22250198 10.1242/jcs.091736

[244] Kheirbek MA, Britt JP, Beeler JA, Ishikawa Y, McGehee DS, Zhuang X. Adenylyl cyclase type 5 contributes to corticostriatal plasticity and striatum-dependent learning. J Neurosci. 2009;29(39):12115–24. 10.1523/JNEUROSCI.3343-09.2009. (**PubMedPMID:19793969;PubMedCentralPMCID:PMC2782774**). 19793969 10.1523/JNEUROSCI.3343-09.2009 PMC2782774

[245] Doyle TB, Muntean BS, Ejendal KF, Hayes MP, Soto-Velasquez M, Martemyanov KA, et al. Identification of novel adenylyl cyclase 5 (AC5) signaling networks in D1 and D2 medium spiny neurons using bimolecular fluorescence complementation screening. Cells. 2019;8(11):1468. 10.3390/cells8111468. 31752385 10.3390/cells8111468 PMC6912275

[246] Hansen JN, Kaiser F, Leyendecker P, Stüven B, Krause J, Derakhshandeh F, et al. A cAMP signalosome in primary cilia drives gene expression and kidney cyst formation. EMBO Rep. 2022;23(8):e54315. 10.15252/embr.202154315. 35695071 10.15252/embr.202154315 PMC9346484

[247] Rahmi U, Goenawan H, Sylviana N, Setiawan I, Putri ST, Andriyani S, et al. Exercise induction at expression immediate early gene (c-Fos, ARC, EGR-1) in the hippocampus: a systematic review. Dement Neuropsychol. 2024;18:e20230015. 10.1590/1980-5764-DN-2023-0015. (**PubMed PMID: 38628561; PubMed Central PMCID: PMC11019719**). 38628561 10.1590/1980-5764-DN-2023-0015 PMC11019719

[248] Gallo FT, Katche C, Morici JF, Medina JH, Weisstaub NV. Immediate early genes, memory and psychiatric disorders: focus on c-Fos, Egr1 and Arc. Front Behav Neurosci. 2018;12:79. 10.3389/fnbeh.2018.00079. (**PubMed PMID: 29755331; PubMed Central PMCID: PMC5932360**). 29755331 10.3389/fnbeh.2018.00079 PMC5932360

[249] Esvald EE, Tuvikene J, Sirp A, Patil S, Bramham CR, Timmusk T. CREB family transcription factors are major mediators of BDNF transcriptional autoregulation in cortical neurons. J Neurosci Off J Soc Neurosci. 2020;40(7):1405–26. 10.1523/JNEUROSCI.0367-19.2019. (**PubMed PMID: 31915257; PubMed Central PMCID: PMC7044735**). 10.1523/JNEUROSCI.0367-19.2019 PMC7044735 31915257

[250] Tulving E. Episodic memory: from mind to brain. Annu Rev Psychol. 2002;53(1):1–25. 10.1146/annurev.psych.53.100901.135114. 11752477 10.1146/annurev.psych.53.100901.135114

[251] Squire LR. Memory systems of the brain: a brief history and current perspective. Neurobiol Learn Mem. 2004;82(3):171–7. 10.1016/j.nlm.2004.06.005. 15464402 10.1016/j.nlm.2004.06.005

[252] Richards BA, Frankland PW. The persistence and transience of memory. Neuron. 2017;94(6):1071–84. 10.1016/j.neuron.2017.04.037. 28641107 10.1016/j.neuron.2017.04.037

[253] Gershman SJ, Fiete I, Irie K. Key-value memory in the brain. Neuron. 2025;113(11):1694-1707.e1. 10.1016/j.neuron.2025.02.029. (**PubMed PMID: 40147436**). 40147436 10.1016/j.neuron.2025.02.029

[254] Buonomano DV, Maass W. State-dependent computations: spatiotemporal processing in cortical networks. Nat Rev Neurosci. 2009;10(2):113–25. 10.1038/nrn2558. 19145235 10.1038/nrn2558

[255] Mante V, Sussillo D, Shenoy KV, Newsome WT. Context-dependent computation by recurrent dynamics in prefrontal cortex. Nature. 2013;503(7474):78–84. 10.1038/nature12742. 24201281 10.1038/nature12742 PMC4121670

[256] Calhoun AJ, Pillow JW, Murthy M. Unsupervised identification of the internal states that shape natural behavior. Nat Neurosci. 2019;22(12):2040–9. 10.1038/s41593-019-0533-x. 31768056 10.1038/s41593-019-0533-x PMC7819718

[257] Silver D, Huang A, Maddison CJ, Guez A, Sifre L, Van Den Driessche G, et al. Mastering the game of Go with deep neural networks and tree search. Nature. 2016;529(7587):484–9. 10.1038/nature16961. 26819042 10.1038/nature16961

[258] Graves A, Wayne G, Reynolds M, Harley T, Danihelka I, Grabska-Barwińska A, et al. Hybrid computing using a neural network with dynamic external memory. Nature. 2016;538(7626):471–6. 10.1038/nature20101. 27732574 10.1038/nature20101

[259] Josselyn SA, Tonegawa S. Memory engrams: recalling the past and imagining the future. Science. 2020;367(6473):eaaw4325. 10.1126/science.aaw4325. (**PubMed PMID: 31896692; PubMed Central PMCID: PMC7577560**). 31896692 10.1126/science.aaw4325 PMC7577560

[260] Nader K. Reconsolidation and the dynamic nature of memory. Cold Spring Harb Perspect Biol. 2015;7(10):a021782. 10.1101/cshperspect.a021782. (**PubMed PMID: 26354895; PubMed Central PMCID: PMC4588064.**). 26354895 10.1101/cshperspect.a021782 PMC4588064

[261] Rashid AJ, Yan C, Mercaldo V, Hsiang HL (Liz), Park S, Cole CJ, et al. Competition between engrams influences fear memory formation and recall. Science. 2016;353(6297):383–7. 10.1126/science.aaf0594. 27463673 10.1126/science.aaf0594 PMC6737336

[262] Sun X, Bernstein MJ, Meng M, Rao S, Sørensen AT, Yao L, et al. Functionally distinct neuronal ensembles within the memory engram. Cell. 2020;181(2):410-423.e17. 10.1016/j.cell.2020.02.055. (**PubMed PMID: 32187527; PubMed Central PMCID: PMC7166195.**). 32187527 10.1016/j.cell.2020.02.055 PMC7166195

[263] Wang Z, Phan T, Storm DR. The type 3 adenylyl cyclase is required for novel object learning and extinction of contextual memory: role of cAMP signaling in primary cilia. J Neurosci. 2011;31(15):5557–61. 10.1523/JNEUROSCI.6561-10.2011. (**PubMed PMID: 21490195.**). 21490195 10.1523/JNEUROSCI.6561-10.2011 PMC3091825

[264] Strobel MR, Zhou Y, Qiu L, Hofer AM, Chen X. Temporal ablation of the ciliary protein IFT88 alters normal brainwave patterns. Sci Rep. 2025;15(1):347. 10.1038/s41598-024-83432-1. 39747370 10.1038/s41598-024-83432-1 PMC11697071

[265] Frey U, Morris RGM. Synaptic tagging and long-term potentiation. Nature. 1997;385(6616):533–6. 10.1038/385533a0. 9020359 10.1038/385533a0

[266] Sajikumar S, Frey JU. Late-associativity, synaptic tagging, and the role of dopamine during LTP and LTD. Neurobiol Learn Mem. 2004;82(1):12–25. 10.1016/j.nlm.2004.03.003. 15183167 10.1016/j.nlm.2004.03.003

[267] Redondo RL, Morris RGM. Making memories last: the synaptic tagging and capture hypothesis. Nat Rev Neurosci. 2011;12(1):17–30. 10.1038/nrn2963. 21170072 10.1038/nrn2963

[268] Shetty MS, Sharma M, Hui NS, Dasgupta A, Gopinadhan S, Sajikumar S. Investigation of synaptic tagging/capture and cross-capture using acute hippocampal slices from rodents. J Vis Exp. 2015;103:53008. 10.3791/53008. (**PubMed PMID: 26381286; PubMed Central PMCID: PMC4692586.**). 10.3791/53008 PMC4692586 26381286

[269] Kandel ER. The molecular biology of memory: cAMP, PKA, CRE, CREB-1, CREB-2, and CPEB. Mol Brain. 2012;5(1):14. 10.1186/1756-6606-5-14. 22583753 10.1186/1756-6606-5-14 PMC3514210

[270] Kandel ER, Dudai Y, Mayford MR. The molecular and systems biology of memory. Cell. 2014;157(1):163–86. 10.1016/j.cell.2014.03.001. (**PubMed PMID: 24679534**). 24679534 10.1016/j.cell.2014.03.001

[271] Alberini CM. Transcription factors in long-term memory and synaptic plasticity. Physiol Rev. 2009;89(1):121–45. 10.1152/physrev.00017.2008. 19126756 10.1152/physrev.00017.2008 PMC3883056

[272] Rossato JI, Bevilaqua LRM, Izquierdo I, Medina JH, Cammarota M. Dopamine controls persistence of long-term memory storage. Science. 2009;325(5943):1017–20. 10.1126/science.1172545. 19696353 10.1126/science.1172545

[273] Wong ST, Trinh K, Hacker B, Chan GCK, Lowe G, Gaggar A, et al. Disruption of the type III adenylyl cyclase gene leads to peripheral and behavioral anosmia in transgenic mice. Neuron. 2000;27(3):487–97. 10.1016/S0896-6273(00)00060-X. 11055432 10.1016/s0896-6273(00)00060-x

[274] Kitamura T, Ogawa SK, Roy DS, Okuyama T, Morrissey MD, Smith LM, et al. Engrams and circuits crucial for systems consolidation of a memory. Science. 2017;356(6333):73–8. 10.1126/science.aam6808. 28386011 10.1126/science.aam6808 PMC5493329

[275] Roy DS, Park YG, Kim ME, Zhang Y, Ogawa SK, DiNapoli N, et al. Brain-wide mapping reveals that engrams for a single memory are distributed across multiple brain regions. Nat Commun. 2022;13(1):1799. 10.1038/s41467-022-29384-4. 35379803 10.1038/s41467-022-29384-4 PMC8980018

[276] Bi G, Poo M. Synaptic modifications in cultured hippocampal neurons: dependence on spike timing, synaptic strength, and postsynaptic cell type. J Neurosci. 1998;18(24):10464–72. 10.1523/JNEUROSCI.18-24-10464.1998. 9852584 10.1523/JNEUROSCI.18-24-10464.1998 PMC6793365

[277] Dan Y, Poo M. Spike timing-dependent plasticity of neural circuits. Neuron. 2004;44(1):23–30. 10.1016/j.neuron.2004.09.007. 15450157 10.1016/j.neuron.2004.09.007

[278] Fries P. Rhythms for cognition: Communication through coherence. Neuron. 2015;88(1):220–35. 10.1016/j.neuron.2015.09.034. 26447583 10.1016/j.neuron.2015.09.034 PMC4605134

[279] Canolty RT, Knight RT. The functional role of cross-frequency coupling. Trends Cogn Sci. 2010;14(11):506–15. 10.1016/j.tics.2010.09.001. (**PubMed PMID: 20932795; PubMed Central PMCID: PMC3359652**). 20932795 10.1016/j.tics.2010.09.001 PMC3359652

[280] Lisman JE, Jensen O. The theta-gamma neural code. Neuron. 2013;77(6):1002–16. 10.1016/j.neuron.2013.03.007. 23522038 10.1016/j.neuron.2013.03.007 PMC3648857

[281] Adhikari A, Topiwala MA, Gordon JA. Synchronized activity between the ventral hippocampus and the medial prefrontal cortex during anxiety. Neuron. 2010;65(2):257–69. 10.1016/j.neuron.2009.12.002. (**PubMed PMID: 20152131**). 20152131 10.1016/j.neuron.2009.12.002 PMC2822726

[282] Chen X, Luo J, Leng Y, Yang Y, Zweifel LS, Palmiter RD, et al. Ablation of Type III adenylyl cyclase in mice causes reduced neuronal activity, altered sleep pattern, and depression-like phenotypes. Biol Psychiatry. 2016;80(11):836–48. 10.1016/j.biopsych.2015.12.012. 26868444 10.1016/j.biopsych.2015.12.012 PMC5972377

[283] Daume J, Kamiński J, Schjetnan AGP, Salimpour Y, Khan U, Kyzar M, et al. Control of working memory by phase–amplitude coupling of human hippocampal neurons. Nature. 2024;629(8011):393–401. 10.1038/s41586-024-07309-z. 38632400 10.1038/s41586-024-07309-z PMC11078732

[284] Keifer E, Gabor R, Richardson JG, Pomeroy J, Haws RM, Tucker RJ. Intellectual functioning in individuals with Bardet-Biedl syndrome. Clin Neuropsychol. 2026;8:1–16. 10.1080/13854046.2026.2648268. 10.1080/13854046.2026.2648268 41947757

[285] Biel M, Wahl-Schott C, Michalakis S, Zong X. Hyperpolarization-activated cation channels: from genes to function. Physiol Rev. 2009;89(3):847–85. 10.1152/physrev.00029.2008. (**PubMed PMID: 19584315**). 19584315 10.1152/physrev.00029.2008

[286] Marr D. Simple memory: a theory for archicortex. Philos Trans R Soc Lond B Biol Sci. 1971;262(841):23–81. 10.1098/rstb.1971.0078. 4399412 10.1098/rstb.1971.0078

[287] Leutgeb JK, Leutgeb S, Moser MB, Moser EI. Pattern separation in the dentate gyrus and CA3 of the hippocampus. Science. 2007;315(5814):961–6. 10.1126/science.1135801. 17303747 10.1126/science.1135801

[288] Yassa MA, Stark CEL. Pattern separation in the hippocampus. Trends Neurosci. 2011;34(10):515–25. 10.1016/j.tins.2011.06.006. 21788086 10.1016/j.tins.2011.06.006 PMC3183227

[289] Witter MP, Wouterlood FG, Naber PA, Van Haeften T. Anatomical organization of the parahippocampal-hippocampal network. Ann N Y Acad Sci. 2000;911(1):1–24. 10.1111/j.1749-6632.2000.tb06716.x. 10911864 10.1111/j.1749-6632.2000.tb06716.x

[290] Clelland CD, Choi M, Romberg C, Clemenson GD, Fragniere A, Tyers P, et al. A functional role for adult hippocampal neurogenesis in spatial pattern separation. Science. 2009;325(5937):210–3. 10.1126/science.1173215. 19590004 10.1126/science.1173215 PMC2997634

[291] Sahay A, Scobie KN, Hill AS, O’Carroll CM, Kheirbek MA, Burghardt NS, et al. Increasing adult hippocampal neurogenesis is sufficient to improve pattern separation. Nature. 2011;472(7344):466–70. 10.1038/nature09817. 21460835 10.1038/nature09817 PMC3084370

[292] Aimone JB, Li Y, Lee SW, Clemenson GD, Deng W, Gage FH. Regulation and function of adult neurogenesis: from genes to cognition. Physiol Rev. 2014;94(4):991–1026. 10.1152/physrev.00004.2014. 25287858 10.1152/physrev.00004.2014 PMC4280160

[293] Temprana SG, Mongiat LA, Yang SM, Trinchero MF, Alvarez DD, Kropff E, et al. Delayed coupling to feedback inhibition during a critical period for the integration of adult-born granule cells. Neuron. 2015;85(1):116–30. 10.1016/j.neuron.2014.11.023. 25533485 10.1016/j.neuron.2014.11.023 PMC4329739

[294] Drew LJ, Kheirbek MA, Luna VM, Denny CA, Cloidt MA, Wu MV, et al. Activation of local inhibitory circuits in the dentate gyrus by adult‐born neurons. Hippocampus. 2016;26(6):763–78. 10.1002/hipo.22557. 26662922 10.1002/hipo.22557 PMC4867135

[295] Pak TK, Carter CS, Zhang Q, Huang SC, Searby C, Hsu Y, et al. A mouse model of Bardet-Biedl syndrome has impaired fear memory, which is rescued by lithium treatment. PLoS Genet. 2021;17(4):e1009484. 10.1371/journal.pgen.1009484. 33886537 10.1371/journal.pgen.1009484 PMC8061871

[296] Guemez-Gamboa A, Coufal NG, Gleeson JG. Primary cilia in the developing and mature brain. Neuron. 2014;82(3):511–21. 10.1016/j.neuron.2014.04.024. (**PubMed PMID: 24811376; PubMed Central PMCID: PMC4104280.**). 24811376 10.1016/j.neuron.2014.04.024 PMC4104280

[297] Madisen L, Zwingman TA, Sunkin SM, Oh SW, Zariwala HA, Gu H, et al. A robust and high-throughput Cre reporting and characterization system for the whole mouse brain. Nat Neurosci. 2010;13(1):133–40. 10.1038/nn.2467. 20023653 10.1038/nn.2467 PMC2840225

[298] Platt RJ, Chen S, Zhou Y, Yim MJ, Swiech L, Kempton HR, et al. CRISPR-Cas9 knockin mice for genome editing and cancer modeling. Cell. 2014;159(2):440–55. 10.1016/j.cell.2014.09.014. 25263330 10.1016/j.cell.2014.09.014 PMC4265475

[299] Shalem O, Sanjana NE, Zhang F. High-throughput functional genomics using CRISPR–Cas9. Nat Rev Genet. 2015;16(5):299–311. 10.1038/nrg3899. 25854182 10.1038/nrg3899 PMC4503232

[300] Weisheit I, Kroeger JA, Malik R, Klimmt J, Crusius D, Dannert A, et al. Detection of deleterious on-target effects after HDR-mediated CRISPR editing. Cell Rep. 2020;31(8):107689. 10.1016/j.celrep.2020.107689. 32460021 10.1016/j.celrep.2020.107689

[301] Hansen JN, Kaiser F, Klausen C, Stüven B, Chong R, Bönigk W, et al. Nanobody-directed targeting of optogenetic tools to study signaling in the primary cilium. Elife. 2020;9:e57907. 10.7554/eLife.57907. 32579112 10.7554/eLife.57907 PMC7338050

[302] Yang J, Dong Y, Liu J, Peng Y, Wang D, Li L, et al. Primary ciliary protein kinase A activity in the prefrontal cortex modulates stress in mice. Neuron. 2025;113(8):1276-1289.e5. 10.1016/j.neuron.2025.02.002. 40056898 10.1016/j.neuron.2025.02.002

[303] Steinmetz NA, Aydin C, Lebedeva A, Okun M, Pachitariu M, Bauza M, et al. Neuropixels 2.0: a miniaturized high-density probe for stable, long-term brain recordings. Science. 2021;372(6539):eabf4588. 10.1126/science.abf4588. 33859006 10.1126/science.abf4588 PMC8244810

[304] Findling C, Hubert F, International Brain Laboratory, Acerbi L, Benson B, Benson J, et al. Brain-wide representations of prior information in mouse decision-making. Nature. 2025;645(8079):192–200. 10.1038/s41586-025-09226-1. 40903597 10.1038/s41586-025-09226-1 PMC12408363

[305] Gallego JA, Perich MG, Miller LE, Solla SA. Neural manifolds for the control of movement. Neuron. 2017;94(5):978–84. 10.1016/j.neuron.2017.05.025. 28595054 10.1016/j.neuron.2017.05.025 PMC6122849

[306] Nieh EH, Schottdorf M, Freeman NW, Low RJ, Lewallen S, Koay SA, et al. Geometry of abstract learned knowledge in the hippocampus. Nature. 2021;595(7865):80–4. 10.1038/s41586-021-03652-7. 34135512 10.1038/s41586-021-03652-7 PMC9549979

[307] Chaudhuri R, Gerçek B, Pandey B, Peyrache A, Fiete I. The intrinsic attractor manifold and population dynamics of a canonical cognitive circuit across waking and sleep. Nat Neurosci. 2019;22(9):1512–20. 10.1038/s41593-019-0460-x. 31406365 10.1038/s41593-019-0460-x

[308] Khona M, Fiete IR. Attractor and integrator networks in the brain. Nat Rev Neurosci. 2022;23(12):744–66. 10.1038/s41583-022-00642-0. 36329249 10.1038/s41583-022-00642-0

[309] Langdon C, Genkin M, Engel TA. A unifying perspective on neural manifolds and circuits for cognition. Nat Rev Neurosci. 2023;24(6):363–77. 10.1038/s41583-023-00693-x. 37055616 10.1038/s41583-023-00693-x PMC11058347

[310] Zhou A, Cao X, Mahaganapathy V, Azaro M, Gwin C, Wilson S, et al. Common genetic risk factors in ASD and ADHD co-occurring families. Hum Genet. 2023;142(2):217–30. 10.1007/s00439-022-02496-z. 36251081 10.1007/s00439-022-02496-z PMC10177627

[311] Gandal MJ, Zhang P, Hadjimichael E, Walker RL, Chen C, Liu S, et al. Transcriptome-wide isoform-level dysregulation in ASD, schizophrenia, and bipolar disorder. Science. 2018;362(6420):eaat8127. 10.1126/science.aat8127. 30545856 10.1126/science.aat8127 PMC6443102

[312] Dixit A, Parnas O, Li B, Chen J, Fulco CP, Jerby-Arnon L, et al. Perturb-Seq: dissecting molecular circuits with scalable single-cell RNA profiling of pooled genetic screens. Cell. 2016;167(7):1853-1866.e17. 10.1016/j.cell.2016.11.038. 27984732 10.1016/j.cell.2016.11.038 PMC5181115

[313] Replogle JM, Saunders RA, Pogson AN, Hussmann JA, Lenail A, Guna A, et al. Mapping information-rich genotype-phenotype landscapes with genome-scale Perturb-seq. Cell. 2022. 10.1016/j.cell.2022.05.013. 35688146 10.1016/j.cell.2022.05.013 PMC9380471

